# The diagnostic yield of intellectual disability: combined whole genome low-coverage sequencing and medical exome sequencing

**DOI:** 10.1186/s12920-020-0726-x

**Published:** 2020-05-19

**Authors:** Jun Wang, Yan Wang, Liwen Wang, Wang Yang Chen, Min Sheng

**Affiliations:** 1 Department of Neurology, Affiliated Children’s Hospital of Capital Institute of Pediatrics, Beijing, 100020 China; 2 Kaiumph Medical Diagnostics Co,Ltd, Beijing, 100102 China

**Keywords:** Intellectual disability, Copy number variation, Single-nucleotide variations, Detection rate, Whole genome low-coverage sequencing, Medical exome sequencing

## Abstract

**Background:**

Intellectual disability (ID) is a heterogeneous neurodevelopmental disorder with a complex genetic underpinning in its etiology. Chromosome microarray (CMA) is recommended as the first-tier diagnostic test for ID due to high detection rate of copy number variation (CNV).

**Methods:**

To identify an appropriate clinical detection scheme for ID in Han Chinese patients, whole genome low-coverage sequencing was performed as the first-tier diagnostic test, and medical exome sequencing (MES) as the second-tier diagnostic test for patients with negative results of CNVs.

**Results:**

A total of 19 pathogenic CNVs in 16/95(16.84%) ID patients and 10 pathogenic single-nucleotide variations (SNVs), including 6 novel mutations in 8/95(8.42%) ID patients were identified on whom no pathogenic CNVs were discovered. The detection rate of CNVs in ID with multiple congenital anomalies (MCA) subgroup was significantly higher than ID with autism spectrum disorders and other IDs subgroups. And the single-nucleotide variations showed a higher occurrence rate in the other IDs subgroup.

**Conclusions:**

There were differences in the diagnostic yields of different variation types among the three ID subgroups. Our findings provided a new perspective on appropriate clinical detection scheme in different ID subgroups based on statistically significant differences among the three ID subgroups. The application of whole genome low-coverage sequencing as the first-tier diagnostic test for ID with MCA subgroup and MES as the first-tier diagnostic test for other ID subgroup was considered as an efficient clinical detection scheme.

## Background

Intellectual disability (ID) is characterized by cognitive impairment and social adjustment caused by brain damage or incomplete development, and this accounted for a prevalence of about 1–3% in the world [1–4]. ID is a complicated neurodevelopmental disorder that might be affected by a series of heterogeneous factors such as environmental factors, genetic factors, idiopathic factors, neonatal sequelae and other diseases [2]. Many studies have been conducted to reveal the genetic etiology of ID and reported the involvement of more than 600 genes and 130 rare copy number variations (CNVs) in the cause of ID [5–9]. However, the causes in up to 50% cases cannot be found due to complex etiological factors and high genetic heterogeneity [7, 10–13].

The well-known variant types for the cause of ID included chromosome number or structural abnormalities, genome-wide microdeletions or microduplications and single gene defects. Chromosome microarray (CMA) has been recommended as the first-tier diagnostic test for ID, and so, CNVs might have the highest positive rate with regard to ID [14, 15]. Most of the previous studies conducted on the investigation of genetic etiologies of ID were based on CMA [16–18]. According to a previous study, chromosome abnormality and genome-wide microdeletion or microduplication accounted for 10–20% of ID [3, 19]. Although CMA has been recommended as the first-tier diagnostic test for ID, it is still limited due to insufficiency in detecting the single gene defects.

With the rapid development and wide-use of next generation sequencing in the clinical diagnostic field, the novel technologies used for genetic research on ID aimed to identify more causative CNVs and genes [7, 20, 21]. Previous studies have shown the involvement of significant parts in ID patients, wherein negative results were obtained after CMA, and included single gene defects. The single gene defects might account for about 10% of ID [7]. With the discovery of more and more novel or candidate genes, it could be even higher. A number of novel ID candidate genes, such as ASH1L, MBOAT7 and TRIO, were identified by using next generation sequencing in patients with negative results after CMA [22–24].

Most of the genetic studies on intellectual disabilities were conducted on European populations. The genetic research studies on ID of Chinese populations were less. And majority of these studies focused on CNVs through CMA [18]. Investigating the distribution of CNVs, proportion of single gene defects, and evaluation of the effects of different diagnostic platforms in Chinese ID populations provided the proof for selecting appropriate clinical genetic diagnostic method in Chinese ID populations. We herein performed whole genome low-coverage sequencing as the first-tier diagnostic test in 95 Chinese ID patients and then applied MES as the second-tier diagnostic test for those patients with negative results of CNVs. Pathogenic CNVs and single gene defects were identified by both clinical phenotype relevance and genetic interpretation. The detection rates of CNVs and single gene defects in our study were compared with those in the previous study. Furthermore, we subdivided ID patients into three different subgroups, ID with multiple congenital anomalies (MCA), ID with autism spectrum disorders (ASD) and other IDs. Comparison of the detection rates of CNVs and single gene defects among the three subgroups provides a new vision on the choice of use of appropriate clinical diagnostic test method in different ID patients.

## Methods

### Subjects and controls

The patients were recruited from the Department of Neurology, Affiliated Children’s Hospital of the Capital Institute of Pediatrics between 2016.01 and 2018.12. Written informed consent was obtained from all parents. All 95 patients (30 females and 65 males) had clinical manifestations of ID. The patients were subdivided into three subgroups according to whether they had MCA or ASD except ID. Of these, 52 patients had ID with MCA; 14 patients had ID with ASD; and 29 patients had other IDs.

### Whole genome low-coverage sequencing

Genomic DNA was extracted from the peripheral blood sample using QIAamp DNA Mini Kit (QIAGEN). The DNA was quantified by using Nanodrop 2000 (Thermal Fisher Scientific, DE). The DNA was sheared to size by approximately 300 bp with a Covaris S2 sonicator according to the Illumina TruSeq DNA protocol. The fragments were then end-repaired and A-tailed in preparation for ligation to adapters. The ligation product was amplified by PCR by using the universal primers. The enriched libraries were sequenced on an Illumina HiSeq 2000 sequencer (Illumina, San Diego, CA, USA) for paired-end reads of 150 bp. High-quality paired-end reads were aligned to the NCBI human reference genome GRCh37 by using Short Oligonucleotide Analysis Package (SOAP) aligner software (SOAP2.21; soap.genomics.org.cn/soapsnp.html) [25]. CNV detection was performed according to a three-step method as reported previously [26, 27]. 1 ~ 2X coverage was chosen and 3 ~ 6Gb are sequenced per patient (Supplemental Data Table S3). The resolution is about 100 ~ 200 kb in this condition referred to previous literatures.

### The CNV interpretation

To evaluate the pathogenicity of CNVs, the criteria of American College of Medical Genetics and Genomic (ACMG) guidelines for CNVs in 2011 were mainly referred [28]. A CNV was evaluated as pathogenic if it complies with one of the following criteria: when 1) it overlaps with at least 50% of the critical regions of the known genomic disorders; 2) it contains known ID disease-causing genes and the variation of dosage effect conforms to the genetic pattern; and 3) the size of the CNV is larger than 1 Mb and the region has been recorded in patients with ID in DECIPHER (DatabasE of genomiC varIation and Phenotype in Humans using Ensembl Resources) (https://decipher.sanger.ac.uk/). CNV was evaluated as an uncertain clinical significant CNV when it was documented in < 0.1% of the population and did not meet the criteria of pathogenic CNVs.

### Medical exome sequencing

Genomic DNA was extracted from the peripheral blood by using QIAamp DNA Mini Kit (QIAGEN). DNA was quantified with Nanodrop 2000 (Thermal Fisher Scientific, DE). A minimum of 3 μg DNA was used for the indexed Illumina libraries according to the manufacturer’s protocol. The DNA fragments with sizes ranging from 350 bp to 450 bp and those including the adapter sequences were selected for DNA libraries. Next, over 4000 genes (Supplemental Data Table S2) associated with monogenic disorders were selected by a gene capture strategy by using a custom enrichment kit (IDT, Coralville, Iowa, USA). The enriched libraries were sequenced on an Illumina HiSeq XTen sequencer (Illumina, San Diego, CA, USA) for paired-end reads of 150 bp. 100 ~ 200X mean coverage was chosen and 4 ~ 6Gb are sequenced per patient (Supplemental Data Table S4). Following sequencing, the raw image files were processed by using Bcl2Fastq software (Bcl2Fastq 2.18.0.12, Illumina, Inc.) for base calling and raw data generation. Low-quality variations were filtered out by a quality score of ≥20. SOAP aligner software (SOAP2.21; soap.genomics.org.cn/soapsnp.html) was then used to align the clean reads with the reference human genome (GRCh37). Polymerase chain reaction (PCR) duplicates were removed by using the Picard program. Subsequently, single nucleotide polymorphisms (SNPs) were determined by using the SOAPsnp program, reads were realigned by using Burrows-Wheeler Aligner software 0.7.15, and the insertions and deletions (InDels) were detected by using Genome Analysis Toolkit (GATK) software 3.7. The identified SNPs and InDels were annotated by using the Exome-assistant program. The pathogenicity of SNPs and InDels was evaluated according to the ACMG guidelines.

## Results

### Rare copy number variations

**Fig. 1:**
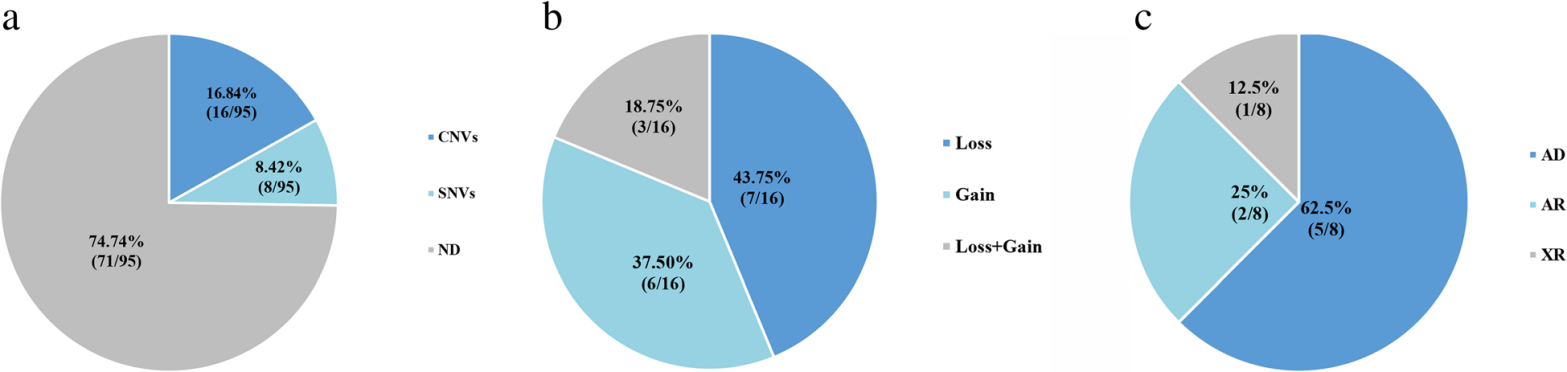
Status of molecular diagnosis after CNV-seq and MES of 95 patients with intellectual disability. **a**) The detection rates of CNVs and SNVs. **b**) The proportion of different CNVs types. **c**) The inheritance patterns of diseases detected by MES

To explore whether the ID was caused by rare CNVs, a whole genome low-coverage sequencing was used as the first-tier diagnostic test in 95 patients diagnosed with ID. As many point variations were SNPs, not all CNVs were rare and implied clinical significance [29]. Therefore, the rare CNVs with population frequency < 0.1% were mainly focused. A total of 312 rare CNVs were identified in 95 patients including 175 gains and 137 losses. The size of the rare CNVs in patients ranged from 102 Kb to 155 Mb. According to the criteria of evaluation of the pathogenicity of rare CNVs in the Methods section, 19 pathogenic CNVs were identified in 16 patients, including 3 patients each carrying both deletion and duplication (Fig. 1a, b, Table 1). Fourteen CNVs (8 losses and 6 gains) were associated with known genomic disorders including 2q31.1 microdeletion syndrome, 7q31 microdeletion syndrome, 7p duplication syndrome, 9p duplication syndrome, 10q deletion syndrome, Jacobsen syndrome, Prader-Willi and Angelman syndrome, Renal cysts and diabetes (RCAD),17p11.2 duplication syndrome, Smith-Mageni syndrome, 2q37 deletion syndrome, and Klinefelter syndrome (Table 1, Patient 1, 3–16). We also identified a 4.43 Mb duplication at 5q35 containing the dosage sensitivity gene *NSD1* that contributed to Sotos syndrome (Table 1, Patient 2). Four CNVs were larger than 1 Mb and the region has been recorded in patients with ID in DECIPHER included a 2.92 Mb deletion at 2q24.1, a 6.36 Mb duplication at 19q13.42–13.43, a 15.04 Mb duplication at 20p12.1-p13 and a 11.66 Mb deletion at 9p23–24.3 (Table 1, Patient 1, 12, 13, 14).

**Table 1:** Pathogenic copy number variations identified by whole genome low-coverage sequencing

Patient	CNV variation	Region size	Known genomic disease	Patient documented in Decipher Database	Patient Phenotype in this study
1	del(2q24.1)(155.48 Mb–158.4 Mb)*1 del(2q31.1-q31.2)(176.16 Mb–178.22 Mb)*1	2.92 Mb 2.06 Mb	2q31.1 microdeletion syndrome	2:154,961,717–158,712,890*1, Pathogenic, 3.75 Mb, Autistic behavior, Clinodactyly of the 5th finger, Cognitive impairment, Intellectual disability, mild, Joint laxity, Kyphoscoliosis, Language impairment, Mandibular prognathia, Muscular hypotonia, Seizures, Synophrys, Thick eyebrow, Tremor 2:156,830,779–159,106,872*1, Unknown, 2.28 Mb, Delayed speech and language development, Intellectual disability, Macrocephaly, Tall stature 2:155,796,265–157,725,469*1, Unknown, 1.93 Mb, Global developmental delay, Intellectual disability, severe	Mental retardation, Abnormal finger, CHD
2	Dup(5q35.2-q35.3)(175.74 Mb–180.08 Mb)*3	4.43 Mb	Sotos syndrome	5:175,714,974–180,696,832*3, Pathogenic, 4.98 Mb, Brachycephaly, Hypertelorism, Microcephaly, Short stature, Unilateral ptosis 5:175,207,164–180,694,002*3, Unknown, 5.49 Mb	Mental retardation, Recurrent respiratory infections, Simiancrease, Limbs Hypotonia
3	del(7q31.1-q31.33)(113.9 Mb–125.52 Mb)*1	11.62 Mb	7q31 microdeletion syndrome	7:114,921,919–126,025,662*1, Unknown, 11.10 Mb, 7:114,236,695–127,881,806*1, Unknown, 13.65 Mb, Delayed speech and language development, Intellectual disability, Muscular hypotonia 7:112,510,560–121,723,279*1,Unknown, 9.21 Mb, Delayed speech and language development, Intellectual disability, Seizures 7:112,137,064–119,186,429*1, Pathogenic, 7.05 Mb, Abnormal facial shape, Moderate global developmental delay	Mental retardation, Asophia, Simiancrease, Facial abnormality
4	dup(7p14.3-p22.3)(0.1 Mb–30.82 Mb)*3	30.82 Mb	7p duplicationsyndrome	7:10,239–25,112,979*3, Unknown, 25.10 Mb, Absent speech, Constipation, Global developmental delay, Intellectual disability, severe, Long fingers, Low-set ears, Micrognathia, Muscular hypotonia, Narrow mouth, Pancreatitis, Thoracolumbar scoliosis 7:503,373–11,090,297*3, Pathogenic, 10.59 Mb, Autism, Broad forehead, Diastema, Generalized joint laxity, Global developmental delay, Micrognathia, Self-injurious behavior, Wide nose 7:109,626–16,317,319*3, Unknown, 16.21 Mb, Abnormality of prenatal development or birth, Cleft palate, Depressed nasal bridge, Low-set ears, Micrognathia	Mental retardation, Poor hearing, Wide set eyes, Simiancrease, Hypotonia, Low set ears, Microphallus, Scrotum, High-vaulted arch
5	dup(9p22.2-p23)(10.06 Mb–16.78 Mb)*3	6.72 Mb	9p duplication syndrome	9:9,910,369–19,437,090*3, Likely pathogenic, 9.53 Mb, Aplasia/Hypoplasia of the distal phalanges of the hand, Aplasia/Hypoplasia of the distal phalanges of the toes, Aplasia/Hypoplasia of the middle phalanges of the hand, Aplasia/Hypoplasia of the middle phalanges of the toes, Cupped ear, Diastasis recti, Epicanthus, Glabellar hemangioma, Hemangioma, Hypertelorism, Low-set ears, Nail dystrophy, Relative macrocephaly 9:8,266,233–16,527,801*3, Unknown, 8.26 Mb, Global developmental delay	Mental retardation, Abnormality of the palmar creases, Laryngomalacia, Muscular hypertonia, Facial abnormality
6	del(10q26.13-q26.3)(126.62 Mb–135.52 Mb) *1	8.9 Mb	10q deletion syndrome	10:126198009–135,430,043*1, Pathogenic, 9.23 Mb (Imbalance arising from a balanced parental rearrangement) 10:127,120,633–135,427,143*1, Likely pathogenic, 8.31 Mb, Congenital strabismus, Constipation, Generalized hypotonia, Language impairment, Microcephaly, Moderate expressive language delay, Sleep-wake cycle disturbance, Temperature instability 10:125,632,306–135,434,148*1, Unknown, 9.80 Mb, Functional abnormality of the bladder, Intellectual disability, moderate, Patent urachus, Strabismus (Imbalance arising from a balanced parental rearrangement)	Mental retardation, Small hands and feet, Hypoplastic labia minora, Hypotonia
7	del(11q24-q25)(124.55 Mb–134.94 Mb)*1	10.39 Mb	Jacobsen syndrome	11:124,205,261–134,868,378*1, Pathogenic, 10.66 Mb, Abnormal platelet count, Epicanthus, Global developmental delay, Hypertelorism, Low-set ears, Short nose, Short stature, Smooth philtrum, Thin upper lip vermilion, Thrombocytopenia	Mental retardation, Facial abnormality, Simiancrease
8	del(15q11.2-q13.2)(23.62 Mb–30.38 Mb)*1	6.76 Mb	Prader-Willi and Angelman syndrome	15:23,619,912–28,438,266*1, Pathogenic, 4.82 Mb, EEG abnormality, Intellectual disability, Microcephaly, Seizures, Truncal ataxia 15:23,619,912–28,438,266*1, Pathogenic, 4.82 Mb, Feeding difficulties in infancy, Hypogonadism, Intellectual disability, Muscular hypotonia, Truncal obesity 15:23,699,760–30,322,138*1, Pathogenic, 6.62 Mb, Absent speech, Global developmental delay, Seizures	Mental retardation, Hypotonia, Microphallus, Scrotum
9	chr17: 34918482–36,408,851*1	1.49 Mb	Renal cysts and diabetes (RCAD)	17:34,815,072–36,215,917*1, Pathogenic, 1.40 Mb, Abnormality of the liver, Diabetes mellitus, Multiple renal cysts 17:34,911,952–36,510,799*1, Pathogenic, 1.60 Mb, Fetal choroid plexus cysts, Multicystic kidney dysplasia	Mental retardation, asophia, and brain dysplasia
10	dup(17p11.2-p12)(14.76 Mb–19.48 Mb)*3	4.72 Mb	17p11.2 duplication syndrome	17:16,773,072–20,222,149*3, Pathogenic, 3.45 Mb, Autism, Hyperactivity, Short attention span, Short stature 17:14,097,915–15,470,903*3, Pathogenic, 1.37 Mb, Abnormality of the motor neurons, Decreased motor nerve conduction velocity, Hypertrophic nerve changes, Impaired pain sensation, Impaired proprioception, Impaired temperature sensation, Impaired vibratory sensation, Pes cavus	Mental retardation, Shrill crying, Poor skin, Hypotonia, Cryptorchidism
11	del(17p11.2)(16.6 Mb–20.5 Mb)*1	3.9 Mb	Smith-Mageni syndrome	17:16,590,776–20,463,301*1, Pathogenic, 3.87 Mb, Brachydactyly, Severe global developmental delay, Specific learning disability	Mental retardation, Dwarfism, Abnormal appearance of skull
12	del(2q37.2–37.3)(235.16 Mb– 243.08 Mb) *1 dup(19q13.42-q13.43)(52.76 Mb– 59.12 Mb)*3	7.92 Mb 6.36 Mb	2q37 deletion syndrome	2:235,875,302–243,041,364*1, Pathogenic, 7.17 Mb, Cognitive impairment, Horseshoe kidney, Postnatal microcephaly, Seizures, Underdeveloped nasal alae 19:51,294,464–56,379,713*3, Likely pathogenic, 5.09 Mb, Intellectual disability, Seizures, Short stature, Specific learning disability 19:53,181,823–59,095,418*3, Unknown, 5.91 Mb, Hypodysplasia of the corpus callosum, Mild global developmental delay, Noncommunicating hydrocephalus 19:53,569,329–59,052,715*3, Unknown, 5.48 Mb, Global developmental delay	Mental retardation, facial dysmorphism and global developmental delay, neonatal hypoglycemia
13	del(2q37.3)(240.70 Mb–243 Mb)*1 dup(20p12.1-p13)(0.1 Mb–15.14 Mb)*3	2.30 Mb 15.04 Mb	2q37 deletion syndrome	2:235,875,302–243,041,364*1, Pathogenic, 7.17 Mb, Cognitive impairment, Horseshoe kidney, Postnatal microcephaly, Seizures, Underdeveloped nasal alae 20:121,521–9,691,972*3, Pathogenic, 9.57 Mb, Intellectual disability, mild, Tall stature 20:121,521–3,783,829*3, Pathogenic Partial, 3.66 Mb, Global developmental delay 20:207,270–5,862,333*3, Unknown, 5.66 Mb, Abnormality of the nervous system, Tremor	Mental retardation, facial dysmorphism and global developmental delay, reproductive system abnormality
14	del(9p24.3-p23)(1 Mb–12.66 Mb)*1 dup(7p22.3-p21.2)(1 Mb–15 Mb)*3	11.66 Mb 14 Mb	9p deletion syndrome 7p duplication syndrome	9:271,257–12,907,826*1, Pathogenic, 12.64 Mb, Abnormality of the eye, Absent speech, Bilateral cryptorchidism, Bilateral talipes equinovarus, Cognitive impairment, Downslanted palpebral fissures, Generalized hypotonia, Hypoplasia of the corpus callosum, Micrognathia, Seizures, Synophrys, Ventricular septal defect 7:109,626–16,317,319*3, Unknown, 13.64 Mb, Deep plantar creases, Frontal bossing, Hydrocephalus, Hypertelorism, Intellectual disability, Midface retrusion, Stenosis of the external auditory canal	Mental retardation, Cheilopalatognathus, CHD, Simiancrease, Low set ears, Blepharophimosis, Hyperspasmia
15	47, XXY	X chromosome	Klinefelter syndrome	X:166,314-155,246,643*3, Pathogenic, 155.08Mb, Delayed speech and language development, Microcephaly, Tetralogy of Fallot	Mental retardation
16	47, XXY	X chromosome	Klinefelter syndrome	X:166,314-155,246,643*3, Pathogenic, 155.08Mb, Delayed speech and language development, Microcephaly, Tetralogy of Fallot	Mental retardation, Autism

### Two concurrent pathogenic CNVs in one patient

**Fig. 2:**
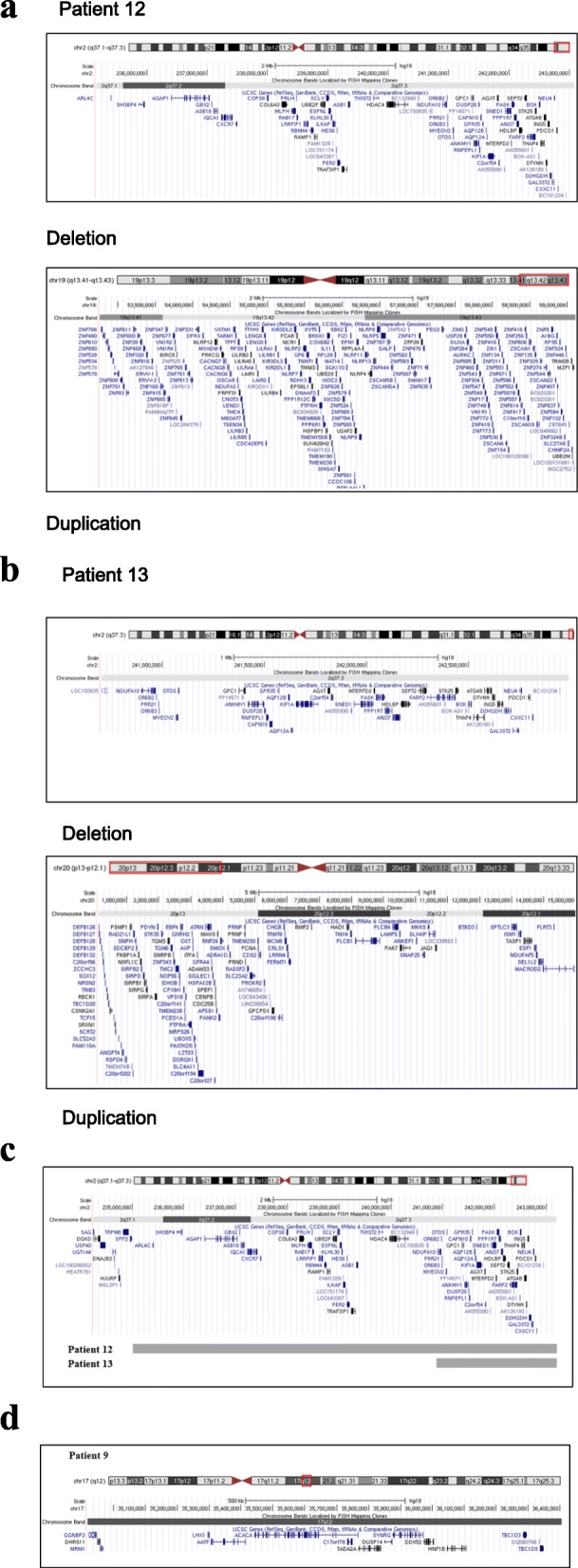
Map of CNVs associated with varying phenotypes of known genomic disorders. **a**) Two concurrent pathogenic CNVs, 2q37.1-q37.3 deletion and 19q13.42-q13.43 duplication, in patient 12. **b**) Two concurrent pathogenic CNVs, 2q37.3 deletion and 20p12.1-p13 duplication, in patient 13. **c**) Map of 2q37 deletions with included genes in 2 patients. and **d**) Map of 17q12 deletions with included genes in patient 9

Cytogenetic imbalances are the most frequently identified causes of ID/MCA. The diagnostic rate of submicroscopic terminal rearrangements was about 6%, with a range of 2 to 29% due to different resolutions of techniques, inclusion criteria and sample sizes [30]. Meanwhile, the frequency of two or more chromosomal aberrations was estimated to be 2–4% by previous studies [31]. Two concurrent pathogenic CNVs were identified by whole genome low-coverage sequencing in 4 of the 16 CNV-positive patients (Table 1, Patient 1, 12, 13, 14), accounting for 25% in CNV-positive patients and 4.2% in the total 95 patients, respectively. The rate was generally consistent with that reported by a previous study based on a large-scale cohort of ID. We identified a 2.06 Mb deletion in 2q31.1 region in 1 patient with a concurrent 2.92 Mb deletion in 2q24.1 region. Patient 14 has a 11.66 Mb deletion in the 7p22.3-p31.2 region and a concurrent 14 Mb duplication in 9p23–24.3 region. Two patients, 12 and 13, carried a deletion in the 2q37 region, with a concurrent 6.36 Mb duplication at 19q13.41 and 15.04 Mb duplication at 20p12.1, respectively (Fig. 2a). Both large deletion and duplication in subtelomeric region were detected in patients 11, 12, and 13, suggesting it as a probably cause of subtelomeric rearrangement. These findings highlighted the importance of screening apparently “balanced” subtelomeric rearrangements inherited from a phenotypically normal parent in patients with ID.

### Variable clinical phenotypes of CNVs

In patient 9, a 1.49 Mb deletion was found at 17q12, and was associated with RCAD, (Fig. 2d). The most consistent clinical feature of RCAD is the presence of renal cysts and most of the affected subjects also had early-onset diabetes. However, Patient 9 in our study showed no signs of renal abnormalities and diabetes until 4 years and 7 months old. Patient 9 showed clinical manifestations of asophia, and brain dysplasia was revealed by MRI instead from the age of 2 years. Interestingly, the recurrent 17q12 deletion has also been documented with diverse range of phenotypes associated with neurodevelopent, such asASD and attention-deficit hyperactivity disorder [32, 33], except RCAD. The 1.49 Mb deletion at 17q12 contains 14 protein-coding genes in addition to HNF1B, which was considered as the etiology of RCAD [34, 35]. This suggested that there might be other protein-coding genes at 17q12 that contributed for the neurodevelopmental disorder, such as *LHX1* and *ACACA*, and were referred to as the genes involved in the neurodevelopmental syndrome [36, 37].

Both patients 12 and 13 carried a deletion in 2q37 region and presented facial dysmorphism and global developmental delay. Some other clinical symptoms were still observed in 2 patients. In addition, a neonatal hypoglycemia was observed in patient 12 and a reproductive system abnormality was observed in patient 13 at 1 years and 4 months. The variable clinical phenotypes between patients 12 and 13 were probably due to different deletion sizes (7.92 Mb vs 2.3 Mb) at 2q37 (Fig. 2c). The other explanation for these was that the additional duplication regions identified in 2 patients 19q13.42q13.43 and 20p12.1–13 (Fig. 2a, b) have been reported with ID and craniofacial dysmorphisms in few case reports [38, 39].

### Single-nucleotide variations

MES was performed for the rest 79 CNV-negative patients. Mutation analysis focused on the genes that previously showed association with ID or related neurodevelopmental disorders. We identified 11 predicted pathogenic variants in 8/95 patients (Table 2). Among the hereditability of patients observed (*n* = 9), autosomal dominant (AD) was most frequent (*n* = 5, 62.5%), followed by autosomal recessive (AR) (*n* = 2, 25%) and X-linked inheritance (*n* = 1, 12.5%) (Fig. 1c). These variants included de novo (n = 5), compounded heterozygous (n = 2), homozygous (n = 1) and hemizygous variants (n = 1). No genes were found to be mutated in two families, suggesting a low incidence of hot spots underlying in ID in Han Chinese population. Compared with the phenotypes of 11 variants in genes that are known to cause ID, most of the patients reported similar phenotypic entities as previous reported.

**Table 2:** Pathogenic or likely pathogenic mutations identified by medical exome sequencing

Patient	Gene	Transcript	Nucleotide change	Amino acid change	Het/Hom	Related disease	origin	Literature report
17	HPRT1	NM_000194	c.419delG	p.Gly140Alafs*26	hemi	Lesch-Nyhan syndrome	maternal	Novel
18	AARS2	NM_020745	c.806 G > A c.374 T > C	p.Gly269Asp p.Leu125Pro	het het	Leukoencephalopathy, progressive, with ovarian failure	Paternal Maternal	Novel Novel
19	EEF1A2	NM_001958	c.796C > T	p.Arg266Trp	het	Intellectual disability, autosomal dominant 38	de novo	Helbig,et al., 2016
20	TCF4	NM_001083962	c.1153C > T	p.Arg385Ter	het	Pitt-Hopkins syndrome	de novo	Zweier,et al., 2007
21	KIF1A	NM_004321	c.1262A > C	p.His421Pro	het	Intellectual disability, autosomal dominant 9	de novo	Novel
22	CACNA1A	NM_001127221	c.4991G > A	p.Arg1664Gln	het	Migraine, familial hemiplegic, 1	de novo	Tonelli,et al., 2006
23	STXBP1	NM_003165	c.536 T > G	p.Leu179Arg	het	Epileptic encephalopathy, early infantile, 4	de novo	Novel
24	B4GALT7	NM_007255	c.319G > C c.614 T > C	p.Glu107Gln p.Leu205Pro	het het	Ehlers-Danlos syndrome, spondylodysplastic type, 1	Paternal Maternal	Novel Okajima,et al., 1999

### Novel mutations identified by medical exome sequencing

We identified 6 novel pathogenic or likely pathogenic single-nucleotide variations in 5/8 ID patients, which included *HPRT1* c.419delG, *AARS2* c.806G > A c.374 T > C, *KIF1A* c.1262A > C, *STXBP1* c.536 T > G and *B4GALT7* c.319G > C (Table 2, Patients 17, 18, 21, 23 and 24). In patient 17, a hemizygous 1-bp deletion (419delG) that caused a frameshift was found at exon 6. Mutations resulted in premature termination of translation of *HPRT* mRNA (p.Gly140Alafs*26), which was not reported previously but a pathogenic missense amino acid change occurred at the same position [40]. A novel compound heterozygous mutation, c.806G > Aand c.374 T > C, was revealed in *AARS2* gene in patient 18 who was presented with psychomotor retardation, hypotonia, cerebellar atrophy and white matter abnormal signal. Both the missense mutations substituted the highly conserved amino acid residues in aminoacylation domain, and were predicted to be damaged by SIFT, Polyphen2 and Mutation Taster. Two novel de novo mutations, *KIF1A* c.1262A > C and *STXBP1* c.536 T > G were identified in patients 21 and 23, respectively. In patient 24, a novel missense mutation c.319G > C caused heterozygous mutation of a novel compound with a reported pathogenic missense mutation at p.Leu205Pro in the *B4GALT7* gene. In addition, we also identified 7 novel variants of uncertain significance in 5 ID patients, which included *AP4M1* c.26C > T, *SLC9A6* c.286G > A, *COX15* c.647C > T c.583G > C, *SOX3* c.316G > A and *CC2D1A* c.270G > C c.2342G > T (Supplemental Data Table S1). The novel variants of uncertain significance needed to be further functional verified.

### Diagnostic yield and variation types in different ID subgroups

**Fig. 3:**
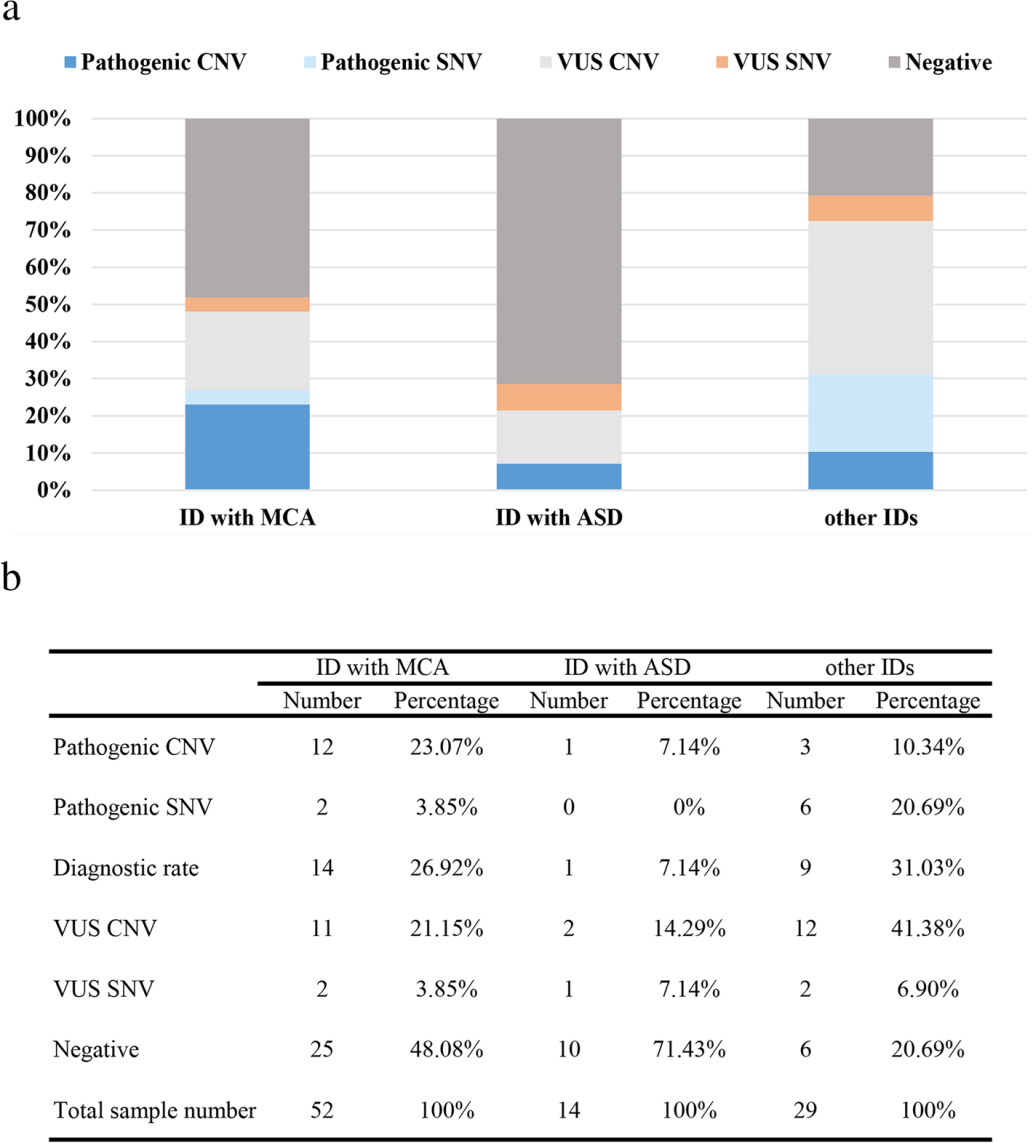
Diagnostic yield in ID cohort (*n* = 95) by subgroup distribution through whole genome low-coverage sequencing and medical exome sequencing. **a**) The histogram of diagnostic rates in different ID cohorts. **b**) The detection result of the patients in different ID cohorts by MES and CNV-seq

The diagnostic yields informed in previous studies of large-scale cohort of IDs were mostly acquired by a mixed ID group that consisted of other phenotypes including MCA and ASD. Previous studies have reported an increased diagnostic yield of CNVs in ID patients with MCA [41, 42]. To investigate whether the diagnostic yields showed differences among different ID subgroups, the ID patients were subdivided into 3 different subgroups, ID with MCA, ID with ASD and other IDs (ID without MCA and ASD). The diagnostic yield varied according to the ID subgroups. The highest diagnostic rate was observed in other ID subgroup (31.03%), followed by ID with MCA (26.92%). The lowest diagnostic rate was observed in ID with ASD subgroup (7.14%), (Fig. 3a, b). Except the diagnostic yield, the variation types were also varied by ID subgroups. In patients with ID, the MCA CNVs accounted for 23.07% and single gene defects accounted for 3.85% (Fig. 3a, b). In other IDs, CNVs accounted for 10.34% and single gene defects accounted for 20.69% (Fig. 3a, b). The rate of ID CNVs in MCA subgroup was significantly higher than the other two subgroups. Likewise, single gene defects contributed to a larger proportion in other ID subgroup. The varied diagnostic yields and variation types suggested that the clinical detection scheme of ID might be changed along with different ID subgroups that patients belong to. These results provide a new perspective on the appropriate clinical detection scheme for different ID subgroups.

## Discussion

A total of 19 pathogenic CNVs in 16.84% (16/95) of Han Chinese ID patients were found by whole genome low-coverage sequencing. This rate was mainly consistent with that of a previous study based on European and Chinese populations by CMA [43–45]. The remaining subtle difference was likely to be due to other factors, such as the technology platform or the criteria of pathogenic CNVs. These results validated the whole genome low-coverage sequencing as an alternative effective diagnostic method for genome-wide CNV detection in routine clinical application [46]. In the clinical diagnosis of pathogenic CNVs, whole genome low-coverage sequencing approach shows equivalent effectiveness and advantages compared with CMA by recent studies. Meanwhile whole genome low-coverage sequencing is a more sensitive, operable, rapid and lower cost method rather than conventional CMA. The only limition of whole genome low-coverage sequencing was unable to detect uniparental disomy compared with CMA. With the development of NGS, whole genome low-coverage sequencing will have a better application prospect compared with conventional CMA.

Due to relatively little realization of the CNVs when compared with single-nucleotide variations, the database of pathogenic CNVs and well-known genome-wide disorders still remained poor. Therefore, there were several CNVs judged as variants but with uncertain significance. Among CNVs that are associated with well-known genome-wide disorders in patients, a 1.49 Mb deletion at 17p11.2 locus was noticed which was reported by RCAD. The 17p11.2 deletion was also been found in a patient characterized by ID, cerebral dysplasia, asophia, big head circumference and slightly lower limb muscle tone. The patient who carried 17p11.2 deletion in our study also presented with some neurological symptoms such as asophia and brain dysplasia. These findings implied the correlation between the 17p11.2 region and neurodevelopmental disorders. Patient 12 and 13 both carried 2q37 deletion. They both had ID and facial dysmorphism, but also showed specific phenotypes themselfs. The diversity in phenotypes might be explained by different boundaries of deletion regions encompassing different genes among different patients or additional modified variations in other areas such as epigenetics [35, 47, 48]. This indicated that the study of pathogenic mechanisms of CNVs were more complicated than SNVs. The genetic contributors for many pathogenic CNVs and even for some well-known disorders such as 16p11.2 microdeletion syndrome were yet to be revealed.

In addition to the CNVs, we applied medical exome sequencing to screen single-nucleotide variations in patients where no pathogenic CNVs were detected. Diagnostic yield showed improvement as the resolution of cytogenetic testing in patients with developmental disabilities has been evolved. A total of 10 pathogenic mutations among 8 genes in 8 unrelated patients were identified. No two patients carried the same disease-causing gene. This indicated a strong genetic heterogeneity and no hot spot genes for ID in Han Chinese population. The disease type caused by the genes varied from syndromic and non-syndromic ID to epileptic encephalopathy, leukoencephalopathy and spastic paraplegia, showing a strong clinical heterogeneity. This might explain the low detection rate (~ 10%) of whole exome sequencing for ID patients as previous study [49].

The patients with multiple congenital anomalies might have a higher detection rate of CNVs than other ID patients [41, 42]. Therefore, the ID patients were subdivided into 3 subgroups, including ID with MCA, ID with ASD and other IDs. The diagnostic yield and variation types were different in different ID subgroups. ID with MCA subgroup showed a higher detection rate of CNVs. ID with ASD showed the lowest detection rate in both CNVs and SNVs. More single-nucleotide variations were identified in other ID subgroups. This suggested that the proportion of variation types were significantly different in different subgroups.

Seventy (74.74%) patients with ID have still not received a molecular diagnosis in our study (Fig. 1a). This was consistent with the results conducted by previous studies where a majority of ID cases remain undiagnosed after genetic detection due to different technology platforms such as microarray, ID panel and whole exome sequencing [7, 10–13]. Such a high negative rate might be caused by lots of factors. The singleton-approach was one the of the main reasons as many studies had showed trio-approach can further improve the detection rate of de-nove mutations in ID cohorts. The trio-approach can also break the limit of lacking parental genotypes. Other factors, caused the high negative rate, were the limitation in resolution of CNVs, the detection area of sequencing and the sensitivity of low frequency mosaic mutation [6]. The resolution of CNVs through whole genome low-coverage sequencing is 100 kb, which meant that the smaller CNVs cannot be detected such as single-exon and intra-exonic deletions. Medical exome sequencing was applied due to its cost-effective detection scheme for SNVs. Meanwhile, the novel genes might be omitted and the mutations outside the coding regions require further exploration. Recent studies have revealed that the low frequency mosaic mutation might play a role partly in neurological and neuromuscular disorders [6, 50].

## Conclusions

Although there were many genetic studies that focused on uncovering the etiology of ID, the information of ID in Han Chinese population was still little. Most of the previous studies were based on CMA. We herein performed whole genome low-coverage sequencing as the first-tier diagnostic test and medical exome sequencing as the second-tier diagnostic test for patients with negative results of CNVs. The detection rates of CNVs and SNVs were consistent with those of the previous studies at home and abroad. This detected scheme was considered appropriate and cost-effective in terms of the consequences. The significantly different proportions of variation types prompted us to select the appropriate detection scheme for different ID subgroups based on the results of this study. However, the sample size of this study is small, and the conclusion might be influenced by sample bias. This study should be conducted in a larger study to eliminate the influence of patient’s source selection, and so, a larger and multicenter study should be conducted. Our study provided a new perspective regarding the study of clinical molecular diagnosis for ID patients in Han Chinese population.

## Supplementary information

**Additional file 1: Supplemental Data Table S1.** Variants of uncertain significance identified by medical exome sequencing.

**Additional file 2: Supplemental Data Table S2.** Gene list of Medical exome sequencing.

**Additional file 3: Supplemental Data Table S3.** The QC of Whole genome low-coverage sequencing.

**Additional file 4: Supplemental Data Table S4.** The QC of Medical exome sequencing.

**Additional file 5: Supplemental Data Table S5.** The clinical phenotype of 95 ID patients.

## Data Availability

NCBI human reference genome GRCh37 was used as the reference genome during the current study, which is available in National Center for Biotechnology Information (NCBI) at https://www.ncbi.nlm.nih.gov/projects/genome/guide/human/index.shtml. We used HGMD professional to estimate whether the reported mutations in this study were novel. HGMD professional is a commercial database and the home page is http://www.hgmd.cf.ac.uk/ac/index.php. The clinical phenotype of patients documented in Decipher Database could acquire from DECIPHER Database (Accession numbers 370540, 254867, 296437, 289790, 252793, 254200, 1301, 1298, 321940, 280316, 286019, 266273, 314487, 280509, 357692, 322997, 270875, 308277, 283607, 402343, 286224, 323760, 296349, 356333, 274515, 280491, 296349, 359646, 295476, 283573, 307049 and 288804). The normal human CNV data used during the current study is available in DGV at http://dgv.tcag.ca/dgv/docs/GRCh37_hg19_variants_2020-02-25.txt. To ensure patient confidentiality, data containing potentially identifiable information was not shared. All data generated or analysed during this study, without identifiable information, is available in this published article and its Supplemental Data Tables.

## Abbreviations

ACMG: American College of Medical Genetics and Genomics
ASD: Autism spectrum disorders
CMA: Chromosome microarray
CNV: Copy number variation
DECIPHER: DatabasE of genomiC varIation and Phenotype in Humans using Ensembl Resources
GATK: Genome Analysis Toolkit
ID: Intellectual disability
InDels: Insertions and Deletions
MCA: Multiple congenital anomalies
MES: Medical exome sequencing
PCR: Polymerase chain reaction
RCAD: Renal cysts and diabetes
SNPs: Single nucleotide polymorphisms
