# Supplementary material for: The diagnostic yield of intellectual disability: combined whole genome low-coverage sequencing and medical exome sequencing
Source: BMC Med Genomics. 2020 May 19;13:70. doi: 10.1186/s12920-020-0726-x (PMC7236547; doi:10.1186/s12920-020-0726-x)
Supplement: Supplementary file 1 — Additional file 1: Supplemental Data Table S1. Variants of uncertain significance identified by medical exome sequencing. [file 12920_2020_726_MOESM1_ESM.docx]

**Supplemental Data Table S1** Variants of uncertain significance identified by medical exome sequencing.

| Patient | Gene | Transcript | Nucleotide change | Amino acid change | Het/Hom | Related disease | origin | Literature report |
| --- | --- | --- | --- | --- | --- | --- | --- | --- |
| 25 | AP4M1 | NM_004722 | c.26C>T | p.Ser9Phe | hom | Spastic paraplegia 50 | Paternal Maternal | Novel |
| 43 | SLC9A6 | NM_006359 | c.286G>A | p.Ala96Thr | hemi | Trimethylaminuria | Maternal | Novel |
| 47 | COX15 | NM_078470 | c.647C>T  c.583G>C | p.Pro216Leu  p.Gly195Arg | het  het | Cardioencephalomyopathy, fatal infantile, due to cytochrome c oxidase deficiency 2; Leigh syndrome due to cytochrome c oxidase deficiency | Paternal  Maternal | Novel  Novel |
| 51 | SOX3 | NM_005634 | c.316G>A | p.Ala106Thr | hemi | Mental retardation, X-linked, with isolated growth hormone deficiency; Panhypopituitarism, X-linked | Maternal | Novel |
| 67 | CC2D1A | NM_017721 | c.270G>C  c.2342G>T | p.Glu90Asp  p.Gly781Val | het  het | Mental retardation, autosomal recessive 3 | Paternal  Maternal | Novel  Novel |
