# Supplementary material for: The diagnostic yield of intellectual disability: combined whole genome low-coverage sequencing and medical exome sequencing
Source: BMC Med Genomics. 2020 May 19;13:70. doi: 10.1186/s12920-020-0726-x (PMC7236547; doi:10.1186/s12920-020-0726-x)
Supplement: Supplementary file 2 — Additional file 2: Supplemental Data Table S2. Gene list of Medical exome sequencing. [file 12920_2020_726_MOESM2_ESM.docx]

**Supplemental Data Table S2.** Gene list of Medical exome sequencing

| A2M | A4GALT | AAAS | AAGAB | AARS | AARS2 | AASS | ABAT |
| --- | --- | --- | --- | --- | --- | --- | --- |
| ABCA1 | ABCA12 | ABCA3 | ABCA4 | ABCA7 | ABCB1 | ABCB11 | ABCB4 |
| ABCB6 | ABCB7 | ABCC11 | ABCC2 | ABCC6 | ABCC8 | ABCC9 | ABCD1 |
| ABCD3 | ABCD4 | ABCG2 | ABCG5 | ABCG8 | ABHD12 | ABHD5 | ABL1 |
| ABO | ACACA | ACAD8 | ACAD9 | ACADM | ACADS | ACADSB | ACADVL |
| ACAN | ACAT1 | ACAT2 | ACD | ACE | ACER3 | ACHE | ACKR1 |
| ACO2 | ACOX1 | ACOX2 | ACP2 | ACP5 | ACP6 | ACPT | ACR |
| ACSF3 | ACSL4 | ACTA1 | ACTA2 | ACTB | ACTC1 | ACTG1 | ACTG2 |
| ACTN1 | ACTN2 | ACTN3 | ACTN4 | ACVR1 | ACVR2B | ACVRL1 | ACY1 |
| AD10 | AD5 | AD6 | AD8 | ADA | ADA2 | ADAM10 | ADAM17 |
| ADAM22 | ADAM9 | ADAMTS10 | ADAMTS13 | ADAMTS17 | ADAMTS18 | ADAMTS2 | ADAMTSL2 |
| ADAMTSL4 | ADAR | ADAT3 | ADCY1 | ADCY10 | ADCY3 | ADCY5 | ADCY6 |
| ADD1 | ADD3 | ADGRE2 | ADGRG1 | ADGRG2 | ADGRG6 | ADGRV1 | ADH1B |
| ADH1C | ADIPOQ | ADK | ADNP | ADRA2B | ADRB1 | ADRB2 | ADRB3 |
| ADSL | ADSSL1 | AEBP1 | AFF2 | AFF4 | AFG3L2 | AFP | AGA |
| AGBL1 | AGBL5 | AGK | AGL | AGPAT2 | AGPS | AGRN | AGRP |
| AGT | AGTR1 | AGXT | AGXT2 | AHCY | AHDC1 | AHI1 | AHSG |
| AICDA | AIFM1 | AIMP1 | AIMP2 | AIP | AIPL1 | AIRE | AK1 |
| AK2 | AK7 | AKAP10 | AKAP9 | AKR1C2 | AKR1C4 | AKR1D1 | AKT1 |
| AKT2 | AKT3 | ALAD | ALAS2 | ALB | ALDH18A1 | ALDH1A3 | ALDH2 |
| ALDH3A2 | ALDH4A1 | ALDH5A1 | ALDH6A1 | ALDH7A1 | ALDOA | ALDOB | ALG1 |
| ALG10 | ALG11 | ALG12 | ALG13 | ALG14 | ALG2 | ALG3 | ALG6 |
| ALG8 | ALG9 | ALK | ALMS1 | ALOX12B | ALOX5 | ALOX5AP | ALOXE3 |
| ALPL | ALS2 | ALX1 | ALX3 | ALX4 | AMACR | AMBN | AMELX |
| AMER1 | AMH | AMHR2 | AMMECR1 | AMN | AMPD1 | AMPD2 | AMPD3 |
| AMT | AMTN | ANG | ANGPTL3 | ANGPTL4 | ANK1 | ANK2 | ANK3 |
| ANKH | ANKLE2 | ANKRD11 | ANKRD26 | ANKS6 | ANLN | ANO10 | ANO3 |
| ANO5 | ANO6 | ANOS1 | ANTXR1 | ANTXR2 | ANXA11 | ANXA5 | AP1S1 |
| AP1S2 | AP1S3 | AP2S1 | AP3B1 | AP3B2 | AP3D1 | AP4B1 | AP4E1 |
| AP4M1 | AP4S1 | AP5Z1 | APC | APC2 | APCDD1 | APCS | APOA1 |
| APOA2 | APOA5 | APOB | APOC2 | APOC3 | APOE | APOL1 | APOL2 |
| APOL4 | APOPT1 | APP | APPL1 | APRT | APTX | AQP1 | AQP2 |
| AQP3 | AQP5 | AQP7 | AR | ARCN1 | ARFGEF2 | ARG1 | ARHGAP26 |
| ARHGAP31 | ARHGDIA | ARHGEF10 | ARHGEF18 | ARHGEF2 | ARHGEF6 | ARHGEF9 | ARID1A |
| ARID1B | ARID2 | ARL13B | ARL2BP | ARL6 | ARL6IP1 | ARMC4 | ARMC5 |
| ARMC9 | ARMS2 | ARNT2 | ARPC1B | ARR3 | ARSA | ARSB | ARSE |
| ART4 | ARV1 | ARX | ASAH1 | ASB10 | ASCC1 | ASCL1 | ASH1L |
| ASIP | ASL | ASNS | ASPA | ASPG | ASPH | ASPM | ASPN |
| ASPSCR1 | ASS1 | ASXL1 | ASXL2 | ASXL3 | ATAD1 | ATAD3A | ATCAY |
| ATF6 | ATG16L1 | ATG5 | ATIC | ATL1 | ATL3 | ATM | ATN1 |
| ATOH7 | ATP13A2 | ATP1A2 | ATP1A3 | ATP1B1 | ATP2A1 | ATP2A2 | ATP2B2 |
| ATP2B3 | ATP2C1 | ATP5A1 | ATP5E | ATP6AP1 | ATP6AP2 | ATP6V0A2 | ATP6V0A4 |
| ATP6V1A | ATP6V1B1 | ATP6V1B2 | ATP6V1E1 | ATP7A | ATP7B | ATP8A2 | ATP8B1 |
| ATPAF2 | ATR | ATRX | ATXN1 | ATXN10 | ATXN2 | ATXN3 | ATXN7 |
| ATXN8 | ATXN8OS | AUH | AURKA | AURKAIP1 | AURKC | AUTS2 | AVP |
| AVPR2 | AXIN1 | AXIN2 | B2M | B3GALNT1 | B3GALNT2 | B3GALT6 | B3GAT3 |
| B3GLCT | B4GALNT1 | B4GALT1 | B4GALT7 | B4GAT1 | B9D1 | B9D2 | BAAT |
| BAG3 | BANF1 | BAP1 | BARD1 | BAX | BBIP1 | BBS1 | BBS10 |
| BBS12 | BBS2 | BBS4 | BBS5 | BBS7 | BBS9 | BCAM | BCAP31 |
| BCHE | BCKDHA | BCKDHB | BCKDK | BCL10 | BCL11A | BCL11B | BCL3 |
| BCL6 | BCL9 | BCO1 | BCOR | BCR | BCS1L | BEAN1 | BEST1 |
| BFSP1 | BFSP2 | BGN | BHLHA9 | BHLHE41 | BICC1 | BICD2 | BIN1 |
| BLK | BLM | BLNK | BLOC1S3 | BLOC1S6 | BLVRA | BMND7 | BMND8 |
| BMP1 | BMP15 | BMP2 | BMP4 | BMPER | BMPR1A | BMPR1B | BMPR2 |
| BMS1 | BOLA3 | BPGM | BPTF | BRAF | BRAT1 | BRCA1 | BRCA2 |
| BRDT | BRF1 | BRIP1 | BRPF1 | BRWD3 | BSCL2 | BSG | BSND |
| BTD | BTK | BTNL2 | BUB1B | BVES | C12orf57 | C12orf65 | C15orf41 |
| C19orf12 | C1GALT1C1 | C1QA | C1QB | C1QBP | C1QC | C1QTNF5 | C1R |
| C1S | C2 | C21orf2 | C21orf59 | C2CD3 | C2orf71 | C3 | C4A |
| C4B | C5 | C6 | C7 | C8A | C8B | C8orf37 | C9 |
| C9orf72 | CA12 | CA2 | CA4 | CA5A | CA8 | CABP2 | CABP4 |
| CACNA1A | CACNA1B | CACNA1C | CACNA1D | CACNA1F | CACNA1G | CACNA1H | CACNA1S |
| CACNA2D4 | CACNB2 | CACNB4 | CACNG2 | CAD | CALCR | CALM1 | CALM2 |
| CALR | CALR3 | CAMK2A | CAMK2B | CAMTA1 | CANT1 | CAPN1 | CAPN10 |
| CAPN3 | CAPN5 | CARD11 | CARD14 | CARD9 | CARS2 | CARTPT | CASK |
| CASP10 | CASP14 | CASP8 | CASQ1 | CASQ2 | CASR | CAST | CAT |
| CATSPER1 | CAV1 | CAV3 | CAVIN1 | CBFB | CBL | CBS | CBX2 |
| CC2D1A | CC2D2A | CCBE1 | CCDC103 | CCDC114 | CCDC115 | CCDC151 | CCDC174 |
| CCDC22 | CCDC28B | CCDC39 | CCDC40 | CCDC50 | CCDC65 | CCDC78 | CCDC8 |
| CCDC88A | CCDC88C | CCL11 | CCL2 | CCL3 | CCL3L1 | CCM2 | CCND1 |
| CCND2 | CCNO | CCR5 | CCT5 | CD151 | CD164 | CD19 | CD207 |
| CD209 | CD244 | CD247 | CD27 | CD2AP | CD320 | CD36 | CD3D |
| CD3E | CD3G | CD4 | CD40 | CD40LG | CD44 | CD46 | CD55 |
| CD59 | CD79A | CD79B | CD81 | CD82 | CD8A | CD96 | CDAN1 |
| CDC14A | CDC42 | CDC45 | CDC6 | CDC73 | CDCA7 | CDH1 | CDH11 |
| CDH15 | CDH23 | CDH3 | CDHR1 | CDK10 | CDK13 | CDK4 | CDK5 |
| CDK5RAP2 | CDK6 | CDKL5 | CDKN1B | CDKN1C | CDKN2A | CDON | CDSN |
| CDT1 | CEACAM16 | CEBPA | CEBPE | CEL | CENPE | CENPF | CENPJ |
| CEP104 | CEP120 | CEP135 | CEP152 | CEP164 | CEP19 | CEP290 | CEP41 |
| CEP55 | CEP57 | CEP63 | CEP78 | CEP83 | CERKL | CERS1 | CERS3 |
| CETP | CFAP43 | CFAP44 | CFAP53 | CFAP69 | CFB | CFC1 | CFD |
| CFH | CFHR1 | CFHR3 | CFHR5 | CFI | CFL2 | CFP | CFTR |
| CHAMP1 | CHAT | CHCHD10 | CHCHD2 | CHD1 | CHD1L | CHD2 | CHD4 |
| CHD7 | CHD8 | CHEK2 | CHI3L1 | CHIC2 | CHIT1 | CHKB | CHM |
| CHMP1A | CHMP2B | CHMP4B | CHN1 | CHNG3 | CHRDL1 | CHRM3 | CHRNA1 |
| CHRNA2 | CHRNA3 | CHRNA4 | CHRNA5 | CHRNB1 | CHRNB2 | CHRND | CHRNE |
| CHRNG | CHST14 | CHST3 | CHST6 | CHST8 | CHSY1 | CHUK | CIB2 |
| CIC | CIDEC | CIITA | CILP | CISD2 | CISH | CIT | CITED2 |
| CKAP2L | CLCF1 | CLCN1 | CLCN2 | CLCN4 | CLCN5 | CLCN7 | CLCNKA |
| CLCNKB | CLDN1 | CLDN10 | CLDN14 | CLDN16 | CLDN19 | CLEC4M | CLEC7A |
| CLIC2 | CLIC5 | CLMP | CLN3 | CLN5 | CLN6 | CLN8 | CLP1 |
| CLPB | CLPP | CLPX | CLRN1 | CLTC | CMPK1 | CNBP | CNGA1 |
| CNGA3 | CNGB1 | CNGB3 | CNKSR2 | CNNM2 | CNNM4 | CNPY3 | CNTN1 |
| CNTN2 | CNTNAP1 | CNTNAP2 | COA5 | COA6 | COA7 | COASY | COCH |
| COG1 | COG2 | COG4 | COG5 | COG6 | COG7 | COG8 | COL10A1 |
| COL11A1 | COL11A2 | COL12A1 | COL13A1 | COL17A1 | COL18A1 | COL1A1 | COL1A2 |
| COL25A1 | COL27A1 | COL2A1 | COL3A1 | COL4A1 | COL4A2 | COL4A3 | COL4A3BP |
| COL4A4 | COL4A5 | COL4A6 | COL5A1 | COL5A2 | COL6A1 | COL6A2 | COL6A3 |
| COL7A1 | COL8A2 | COL9A1 | COL9A2 | COL9A3 | COLEC10 | COLEC11 | COLQ |
| COMP | COMT | COPA | COPB2 | COQ2 | COQ4 | COQ6 | COQ7 |
| COQ8A | COQ8B | COQ9 | CORIN | CORO1A | COX10 | COX14 | COX15 |
| COX20 | COX4I2 | COX6A1 | COX6B1 | COX7B | COX8A | CP | CPA6 |
| CPAMD8 | CPLANE1 | CPLX1 | CPN1 | CPOX | CPS1 | CPT1A | CPT1C |
| CPT2 | CR1 | CR2 | CRADD | CRAT | CRB1 | CRB2 | CRBN |
| CREB1 | CREBBP | CRELD1 | CRIPT | CRLF1 | CRNN | CRTAP | CRX |
| CRY1 | CRYAA | CRYAB | CRYBA1 | CRYBA2 | CRYBA4 | CRYBB1 | CRYBB2 |
| CRYBB3 | CRYGB | CRYGC | CRYGD | CRYGS | CRYM | CSF1R | CSF2RA |
| CSF2RB | CSF3R | CSNK1D | CSNK2A1 | CSPP1 | CSRP3 | CST3 | CSTA |
| CSTB | CTBP1 | CTC1 | CTCF | CTDP1 | CTH | CTHRC1 | CTLA4 |
| CTNNA1 | CTNNA3 | CTNNB1 | CTNND1 | CTNS | CTPS1 | CTRC | CTSA |
| CTSC | CTSD | CTSF | CTSK | CUBN | CUL3 | CUL4B | CUL7 |
| CWC27 | CWF19L1 | CX3CR1 | CXCL12 | CXCR1 | CXCR4 | CXorf56 | CYB5A |
| CYB5R3 | CYBA | CYBB | CYC1 | CYCS | CYFIP2 | CYLD | CYP11A1 |
| CYP11B1 | CYP11B2 | CYP17A1 | CYP19A1 | CYP1B1 | CYP21A2 | CYP24A1 | CYP26B1 |
| CYP26C1 | CYP27A1 | CYP27B1 | CYP2A6 | CYP2B6 | CYP2C19 | CYP2C8 | CYP2C9 |
| CYP2D6 | CYP2R1 | CYP2U1 | CYP3A5 | CYP4F22 | CYP4V2 | CYP7B1 | D2HGDH |
| DAB1 | DACT1 | DAG1 | DAOA | DARS | DARS2 | DBH | DBT |
| DCAF17 | DCAF8 | DCC | DCDC2 | DCHS1 | DCLRE1C | DCN | DCPS |
| DCTN1 | DCX | DCXR | DDB2 | DDC | DDHD1 | DDHD2 | DDIT3 |
| DDOST | DDR2 | DDRGK1 | DDX11 | DDX3X | DDX41 | DDX58 | DDX59 |
| DEAF1 | DEC1 | DEK | DENND5A | DEPDC5 | DES | DFFB | DGAT1 |
| DGKE | DGUOK | DHCR24 | DHCR7 | DHDDS | DHFR | DHH | DHODH |
| DHS6S1 | DHTKD1 | DHX30 | DIABLO | DIAPH1 | DIAPH2 | DIAPH3 | DICER1 |
| DIP2B | DIRC2 | DIS3L2 | DISC1 | DISC2 | DKC1 | DLAT | DLC1 |
| DLD | DLEC1 | DLEU2L | DLEU7 | DLG3 | DLL3 | DLL4 | DLX3 |
| DLX4 | DLX5 | DMD | DMGDH | DMP1 | DMPK | DMXL2 | DNA2 |
| DNAAF1 | DNAAF2 | DNAAF3 | DNAAF4 | DNAAF5 | DNAH1 | DNAH11 | DNAH5 |
| DNAI1 | DNAI2 | DNAJB13 | DNAJB2 | DNAJB6 | DNAJC12 | DNAJC19 | DNAJC21 |
| DNAJC3 | DNAJC5 | DNAJC6 | DNAL1 | DNAL4 | DNASE1 | DNASE1L3 | DNM1 |
| DNM1L | DNM2 | DNMT1 | DNMT3A | DNMT3B | DOCK2 | DOCK6 | DOCK7 |
| DOCK8 | DOK7 | DOLK | DONSON | DPAGT1 | DPF2 | DPH1 | DPM1 |
| DPM2 | DPM3 | DPP6 | DPY19L2 | DPYD | DPYS | DRAM2 | DRC1 |
| DRD3 | DRD4 | DRD5 | DSC2 | DSC3 | DSE | DSG1 | DSG2 |
| DSG4 | DSP | DSPP | DST | DSTYK | DTNA | DTNBP1 | DUOX2 |
| DUOXA2 | DUSP6 | DVL1 | DVL3 | DYM | DYNC1H1 | DYNC2H1 | DYNC2LI1 |
| DYRK1A | DYRK1B | DYSF | DZIP1L | EARS2 | EBF3 | EBP | ECE1 |
| ECEL1 | ECHS1 | ECM1 | EDA | EDAR | EDARADD | EDC3 | EDN1 |
| EDN3 | EDNRA | EDNRB | EED | EEF1A2 | EEF2 | EFEMP1 | EFEMP2 |
| EFHC1 | EFL1 | EFNB1 | EFTUD2 | EGF | EGFR | EGLN1 | EGR2 |
| EHBP1 | EHHADH | EHMT1 | EIF2AK3 | EIF2AK4 | EIF2B1 | EIF2B2 | EIF2B3 |
| EIF2B4 | EIF2B5 | EIF2S3 | EIF4A3 | EIF4E | EIF4G1 | ELAC2 | ELANE |
| ELAVL4 | ELMO2 | ELMOD3 | ELN | ELOVL4 | ELOVL5 | ELP1 | ELP2 |
| ELP4 | EMC1 | EMD | EMG1 | EML1 | EMP2 | EMX2 | ENAM |
| ENG | ENO1 | ENO3 | ENPP1 | ENTPD1 | EOGT | EP300 | EPAS1 |
| EPB41 | EPB41L1 | EPB42 | EPCAM | EPG5 | EPHA2 | EPHB2 | EPHB4 |
| EPHX1 | EPHX2 | EPM2A | EPO | EPOR | EPRS | EPS8 | EPS8L2 |
| EPX | ERAL1 | ERBB2 | ERBB3 | ERBB4 | ERCC1 | ERCC2 | ERCC3 |
| ERCC4 | ERCC5 | ERCC6 | ERCC6L2 | ERCC8 | ERF | ERGIC1 | ERLIN1 |
| ERLIN2 | ERMAP | ERMARD | ESCO2 | ESPN | ESR1 | ESRP1 | ESRRB |
| ETFA | ETFB | ETFDH | ETHE1 | ETV6 | EVC | EVC2 | EWSR1 |
| EXOSC2 | EXOSC3 | EXOSC8 | EXPH5 | EXT1 | EXT2 | EXTL3 | EYA1 |
| EYA4 | EYS | EZH2 | F10 | F11 | F12 | F13A1 | F13B |
| F2 | F5 | F7 | F8 | F9 | FA2H | FAAH | FADD |
| FAH | FAM111A | FAM111B | FAM126A | FAM161A | FAM20A | FAM20C | FAM58A |
| FAM83H | FAN1 | FANCA | FANCB | FANCC | FANCD2 | FANCE | FANCF |
| FANCG | FANCI | FANCL | FANCM | FAR1 | FARS2 | FARSB | FAS |
| FASLG | FASTKD2 | FAT2 | FAT4 | FBLN1 | FBLN5 | FBN1 | FBN2 |
| FBP1 | FBXL4 | FBXO31 | FBXO38 | FBXO7 | FCGR1A | FCGR2A | FCGR2B |
| FCGR2C | FCGR3A | FCN3 | FDPS | FDXR | FECH | FERMT1 | FERMT3 |
| FEZF1 | FFAR4 | FGA | FGB | FGD1 | FGD4 | FGF10 | FGF12 |
| FGF14 | FGF16 | FGF17 | FGF20 | FGF23 | FGF3 | FGF5 | FGF8 |
| FGF9 | FGFR1 | FGFR1OP | FGFR2 | FGFR3 | FGG | FH | FHL1 |
| FIBP | FIG4 | FIGLA | FKBP10 | FKBP14 | FKBP5 | FKRP | FKTN |
| FLAD1 | FLCN | FLG | FLI1 | FLNA | FLNB | FLNC | FLRT3 |
| FLT3 | FLT4 | FLVCR1 | FLVCR2 | FMN2 | FMO3 | FMO5 | FMR1 |
| FN1 | FOLR1 | FOXC1 | FOXC2 | FOXD3 | FOXE1 | FOXE3 | FOXF1 |
| FOXG1 | FOXI1 | FOXL2 | FOXN1 | FOXP1 | FOXP2 | FOXP3 | FOXRED1 |
| FRAS1 | FREM1 | FREM2 | FRMD4A | FRMD7 | FRMPD4 | FRRS1L | FRZB |
| FSCN2 | FSHB | FSHR | FTCD | FTH1 | FTL | FTO | FTSJ1 |
| FUCA1 | FUS | FUT1 | FUT2 | FUT6 | FUT8 | FUZ | FXN |
| FXYD2 | FYB | FYCO1 | FZD4 | FZD6 | G6PC | G6PC3 | G6PD |
| GAA | GAB1 | GABBR2 | GABRA1 | GABRA2 | GABRB1 | GABRB2 | GABRB3 |
| GABRD | GABRG2 | GAD1 | GAL | GALC | GALE | GALK1 | GALNS |
| GALNT12 | GALNT3 | GALT | GAMT | GAN | GANAB | GARS | GAS8 |
| GATA1 | GATA2 | GATA3 | GATA4 | GATA5 | GATA6 | GATAD1 | GATAD2B |
| GATM | GBA | GBA2 | GBE1 | GCDH | GCGR | GCH1 | GCK |
| GCKR | GCLC | GCLM | GCM2 | GCNT2 | GCSH | GDAP1 | GDF1 |
| GDF10 | GDF11 | GDF2 | GDF3 | GDF5 | GDF6 | GDF7 | GDF9 |
| GDI1 | GDNF | GEMIN4 | GFAP | GFER | GFI1 | GFI1B | GFM1 |
| GFPT1 | GGCX | GGT1 | GGT2 | GH1 | GHR | GHRHR | GHRL |
| GHSR | GIF | GIGYF2 | GINS1 | GIPC3 | GIPR | GJA1 | GJA3 |
| GJA5 | GJA8 | GJB1 | GJB2 | GJB3 | GJB4 | GJB6 | GJC2 |
| GK | GLA | GLB1 | GLCCI1 | GLDC | GLDN | GLE1 | GLI2 |
| GLI3 | GLIS2 | GLIS3 | GLMN | GLRA1 | GLRB | GLRX5 | GLUD1 |
| GLUD2 | GLUL | GLYCTK | GM2A | GMNN | GMPPA | GMPPB | GNA11 |
| GNA12 | GNAI2 | GNAI3 | GNAL | GNAO1 | GNAQ | GNAS | GNAS-AS1 |
| GNAT1 | GNAT2 | GNB1 | GNB3 | GNB4 | GNB5 | GNE | GNMT |
| GNPAT | GNPTAB | GNPTG | GNRH1 | GNRHR | GNS | GORAB | GOSR2 |
| GOT1 | GP1BA | GP1BB | GP6 | GP9 | GPAA1 | GPC3 | GPC6 |
| GPD1 | GPD1L | GPD2 | GPHN | GPI | GPIHBP1 | GPNMB | GPR101 |
| GPR143 | GPR179 | GPR68 | GPR88 | GPR89B | GPSM2 | GPT2 | GPX1 |
| GPX4 | GREB1L | GREM2 | GRHL2 | GRHL3 | GRHPR | GRIA3 | GRIA4 |
| GRID2 | GRIK2 | GRIN1 | GRIN2A | GRIN2B | GRIN2D | GRIP1 | GRK1 |
| GRM1 | GRM6 | GRN | GRXCR1 | GRXCR2 | GSC | GSDME | GSN |
| GSS | GSTZ1 | GTF2E2 | GTF2H5 | GTPBP2 | GTPBP3 | GUCA1A | GUCA1B |
| GUCY1A3 | GUCY2C | GUCY2D | GUF1 | GUSB | GYG1 | GYPA | GYPB |
| GYPC | GYS1 | GYS2 | GZF1 | H19 | H6PD | HAAO | HABP2 |
| HACE1 | HADH | HADHA | HADHB | HAGH | HAL | HAMP | HARS |
| HARS2 | HAX1 | HBA1 | HBA2 | HBB | HBG1 | HBG2 | HCCS |
| HCFC1 | HCN1 | HCN4 | HCRT | HDAC6 | HDAC8 | HDC | HECW2 |
| HELLS | HEPACAM | HERC1 | HERC2 | HES7 | HESX1 | HEXA | HEXB |
| HFE | HFE2 | HFM1 | HGD | HGF | HGSNAT | HIBCH | HIKESHI |
| HINT1 | HIP1 | HIST1H1E | HIVEP2 | HJV | HK1 | HLA-A | HLA-B |
| HLA-C | HLA-DQA1 | HLA-DQB1 | HLA-DRB1 | HLA-G | HLCS | HMBS | HMCN1 |
| HMGA1 | HMGA2 | HMGB3 | HMGCL | HMGCS2 | HMMR | HMOX1 | HMX1 |
| HNF1A | HNF1B | HNF4A | HNMT | HNRNPA1 | HNRNPA2B1 | HNRNPDL | HNRNPH2 |
| HNRNPK | HNRNPU | HNRPA2B1 | HOGA1 | HOMER2 | HOXA1 | HOXA11 | HOXA13 |
| HOXA2 | HOXB1 | HOXC13 | HOXD10 | HOXD13 | HP | HPC3 | HPC4 |
| HPC5 | HPC6 | HPCA | HPCQTL19 | HPD | HPGD | HPRT1 | HPS1 |
| HPS3 | HPS4 | HPS5 | HPS6 | HPSE2 | HR | HRAS | HRG |
| HS6ST1 | HSD11B1 | HSD11B2 | HSD17B10 | HSD17B3 | HSD17B4 | HSD3B2 | HSD3B7 |
| HSF4 | HSPA9 | HSPB1 | HSPB3 | HSPB8 | HSPD1 | HSPG2 | HTR1A |
| HTR2A | HTRA1 | HTRA2 | HTT | HUWE1 | HYAL1 | HYDIN | HYLS1 |
| IARS | IARS2 | IBA57 | ICAM1 | ICAM4 | ICK | ICOS | IDH1 |
| IDH2 | IDH3B | IDS | IDUA | IER3IP1 | IFIH1 | IFITM3 | IFITM5 |
| IFNAR2 | IFNG | IFNGR1 | IFNGR2 | IFNL3 | IFT122 | IFT140 | IFT172 |
| IFT27 | IFT43 | IFT52 | IFT57 | IFT74 | IFT80 | IFT81 | IGBP1 |
| IGF1 | IGF1R | IGF2 | IGF2BP2 | IGF2R | IGFALS | IGFBP7 | IGHM |
| IGHMBP2 | IGKC | IGLL1 | IGSF1 | IGSF3 | IHH | IKBKB | IKBKG |
| IKZF1 | IL10 | IL10RA | IL10RB | IL11RA | IL12B | IL12RB1 | IL13 |
| IL17F | IL17RA | IL17RC | IL17RD | IL1B | IL1RAPL1 | IL1RN | IL21 |
| IL21R | IL23R | IL2RA | IL2RG | IL31RA | IL36RN | IL4R | IL6 |
| IL6R | IL7R | ILDR1 | IMPA1 | IMPAD1 | IMPDH1 | IMPDH2 | IMPG1 |
| IMPG2 | INF2 | ING1 | INPP5E | INPP5K | INPPL1 | INS | INSL3 |
| INSR | INTU | INVS | IQCB1 | IQCE | IQSEC2 | IRAK3 | IRAK4 |
| IRF1 | IRF2BP2 | IRF3 | IRF4 | IRF5 | IRF6 | IRF7 | IRF8 |
| IRGM | IRS1 | IRS2 | IRX5 | ISCA1 | ISCA2 | ISCU | ISG15 |
| ISPD | ITCH | ITGA2 | ITGA2B | ITGA3 | ITGA6 | ITGA7 | ITGA8 |
| ITGAM | ITGB2 | ITGB3 | ITGB4 | ITGB6 | ITIH4 | ITK | ITM2B |
| ITPA | ITPKC | ITPR1 | ITPR2 | ITPR3 | IVD | IYD | JAG1 |
| JAGN1 | JAK2 | JAK3 | JAM3 | JPH1 | JPH2 | JPH3 | JUP |
| KALRN | KANK1 | KANK2 | KANSL1 | KARS | KAT6A | KAT6B | KATNB1 |
| KBTBD13 | KCNA1 | KCNA2 | KCNA5 | KCNB1 | KCNC1 | KCNC3 | KCND3 |
| KCNE1 | KCNE2 | KCNE3 | KCNH1 | KCNH2 | KCNJ1 | KCNJ10 | KCNJ11 |
| KCNJ13 | KCNJ18 | KCNJ2 | KCNJ5 | KCNJ6 | KCNK18 | KCNK3 | KCNK9 |
| KCNMA1 | KCNMB1 | KCNN4 | KCNQ1 | KCNQ2 | KCNQ3 | KCNQ4 | KCNQ5 |
| KCNT1 | KCNT2 | KCNV2 | KCTD1 | KCTD17 | KCTD7 | KDF1 | KDM1A |
| KDM5C | KDM6A | KDR | KDSR | KEL | KERA | KHDC3L | KHK |
| KIAA0556 | KIAA0586 | KIAA0753 | KIAA1109 | KIDINS220 | KIF11 | KIF14 | KIF1A |
| KIF1B | KIF1BP | KIF1C | KIF21A | KIF22 | KIF2A | KIF4A | KIF5A |
| KIF5C | KIF7 | KIR3DL1 | KISS1 | KISS1R | KIT | KITLG | KIZ |
| KL | KLC2 | KLF1 | KLF11 | KLF6 | KLHDC8B | KLHL10 | KLHL15 |
| KLHL24 | KLHL3 | KLHL40 | KLHL41 | KLHL7 | KLK1 | KLK4 | KLKB1 |
| KLLN | KMT2A | KMT2B | KMT2C | KMT2D | KMT5B | KNG1 | KNL1 |
| KPTN | KRAS | KREMEN1 | KRIT1 | KRT1 | KRT10 | KRT12 | KRT13 |
| KRT14 | KRT16 | KRT17 | KRT18 | KRT2 | KRT25 | KRT3 | KRT4 |
| KRT5 | KRT6A | KRT6B | KRT6C | KRT71 | KRT74 | KRT75 | KRT8 |
| KRT81 | KRT83 | KRT85 | KRT86 | KRT9 | KY | KYNU | L1CAM |
| L2HGDH | LAGE3 | LAMA1 | LAMA2 | LAMA3 | LAMA4 | LAMB1 | LAMB2 |
| LAMB3 | LAMC2 | LAMC3 | LAMP2 | LAMTOR2 | LARGE1 | LARP7 | LARS |
| LARS2 | LAS1L | LAT | LBR | LCA5 | LCAT | LCK | LCT |
| LDB3 | LDHA | LDHB | LDLR | LDLRAP1 | LEMD2 | LEMD3 | LEP |
| LEPR | LEPROT | LFNG | LGALS2 | LGI1 | LGI4 | LGR4 | LHB |
| LHCGR | LHFPL5 | LHX3 | LHX4 | LIAS | LIFR | LIG4 | LIM2 |
| LIMS2 | LINS1 | LIPA | LIPC | LIPE | LIPH | LIPI | LIPN |
| LIPT1 | LIPT2 | LITAF | LMAN1 | LMAN2L | LMBR1 | LMBRD1 | LMF1 |
| LMNA | LMNB1 | LMNB2 | LMO1 | LMO2 | LMOD3 | LMX1B | LOC728989 |
| LONP1 | LOR | LOX | LOXHD1 | LOXL1 | LPAR6 | LPIN1 | LPIN2 |
| LPL | LPP | LRAT | LRBA | LRIG2 | LRIT3 | LRMDA | LRP1 |
| LRP2 | LRP4 | LRP5 | LRP6 | LRP8 | LRPAP1 | LRPPRC | LRRC6 |
| LRRC8A | LRRK2 | LRSAM1 | LRTOMT | LSS | LTA | LTBP2 | LTBP3 |
| LTBP4 | LTC4S | LYL1 | LYRM4 | LYRM7 | LYST | LYZ | LZTFL1 |
| LZTR1 | LZTS1 | MAB21L2 | MAD1L1 | MAD2L2 | MADD | MAF | MAFA |
| MAFB | MAG | MAGED2 | MAGEL2 | MAGI2 | MAGI3 | MAGT1 | MAK |
| MALT1 | MAMLD1 | MAN1B1 | MAN2B1 | MANBA | MAOA | MAP2K1 | MAP2K2 |
| MAP3K1 | MAP3K20 | MAP3K7 | MAP3K8 | MAPK8IP1 | MAPKAPK3 | MAPKBP1 | MAPRE2 |
| MAPT | MARS | MARS2 | MARVELD2 | MASP1 | MASP2 | MAT1A | MATN3 |
| MATR3 | MAX | MB | MBD5 | MBL2 | MBOAT7 | MBTPS2 | MC1R |
| MC2R | MC3R | MC4R | MCC | MCCC1 | MCCC2 | MCEE | MCFD2 |
| MCM2 | MCM4 | MCM5 | MCM6 | MCM8 | MCM9 | MCOLN1 | MCPH1 |
| MDD1 | MDD2 | MDH2 | MDM2 | MECOM | MECP2 | MECR | MED12 |
| MED13L | MED17 | MED23 | MED25 | MEF2A | MEF2C | MEFV | MEGF10 |
| MEGF8 | MEIOB | MEIS2 | MEN1 | MEOX1 | MERTK | MESP2 | MET |
| METTL13 | METTL23 | MFAP5 | MFF | MFHAS1 | MFN2 | MFRP | MFSD2A |
| MFSD8 | MGAT2 | MGME1 | MGP | MIAT | MIB1 | MICU1 | MID1 |
| MID2 | MIF | MIP | MIPEP | MIR17HG | MIR184 | MIR204 | MIR2861 |
| MIR96 | MITF | MKKS | MKRN3 | MKS1 | MLC1 | MLF1 | MLH1 |
| MLH3 | MLLT10 | MLLT11 | MLPH | MLYCD | MMAA | MMAB | MMACHC |
| MMADHC | MMD2 | MME | MMP1 | MMP13 | MMP14 | MMP19 | MMP2 |
| MMP20 | MMP21 | MMP23B | MMP3 | MMP9 | MN1 | MNX1 | MOCOS |
| MOCS1 | MOCS2 | MOG | MOGS | MORC2 | MPC1 | MPDU1 | MPDZ |
| MPI | MPIG6B | MPL | MPLKIP | MPO | MPV17 | MPZ | MRAP |
| MRAP2 | MRE11 | MRPL3 | MRPL44 | MRPS16 | MRPS2 | MRPS22 | MRPS34 |
| MRPS7 | MRTO4 | MS4A1 | MS4A2 | MSH2 | MSH3 | MSH5 | MSH6 |
| MSMB | MSMO1 | MSN | MSR1 | MSRB1 | MSRB3 | MST1R | MSTN |
| MSTO1 | MSX1 | MSX2 | MTAP | MTFMT | MTHFD1 | MTHFR | MTM1 |
| MTMR14 | MTMR2 | MTNR1B | MTO1 | MTOR | MTPAP | MTR | MTRR |
| MTTP | MUC1 | MUC5B | MUC7 | MUSK | MUT | MUTYH | MVD |
| MVK | MXI1 | MYBPC1 | MYBPC3 | MYC | MYCN | MYD88 | MYF6 |
| MYH11 | MYH14 | MYH2 | MYH3 | MYH6 | MYH7 | MYH8 | MYH9 |
| MYL2 | MYL3 | MYL4 | MYLK | MYLK2 | MYMK | MYO15A | MYO18B |
| MYO1E | MYO3A | MYO5A | MYO5B | MYO6 | MYO7A | MYO9B | MYOC |
| MYOT | MYOZ2 | MYPN | MYT1L | NAA10 | NAA15 | NACC1 | NADK2 |
| NAGA | NAGLU | NAGS | NALCN | NANOS1 | NANS | NARS2 | NAT2 |
| NAT8L | NAXE | NBAS | NBEAL2 | NBN | NBPF10 | NBPF11 | NBPF12 |
| NBPF13P | NBPF20 | NBPF25P | NCAPD2 | NCAPD3 | NCAPH | NCF1 | NCF2 |
| NCF4 | NCR3 | NCSTN | NDE1 | NDN | NDP | NDRG1 | NDST1 |
| NDUFA1 | NDUFA10 | NDUFA11 | NDUFA12 | NDUFA13 | NDUFA2 | NDUFA9 | NDUFAF1 |
| NDUFAF2 | NDUFAF3 | NDUFAF4 | NDUFAF5 | NDUFAF6 | NDUFB11 | NDUFB3 | NDUFB9 |
| NDUFS1 | NDUFS2 | NDUFS3 | NDUFS4 | NDUFS6 | NDUFS7 | NDUFS8 | NDUFV1 |
| NDUFV2 | NEB | NECAP1 | NECTIN1 | NECTIN4 | NEDD4L | NEFH | NEFL |
| NEK1 | NEK2 | NEK8 | NEK9 | NEU1 | NEUROD1 | NEUROG3 | NEXMIF |
| NEXN | NF1 | NF2 | NFE2L2 | NFIA | NFIX | NFKB1 | NFKB2 |
| NFKBIA | NFKBIA? | NFKBIL1 | NFU1 | NGF | NGLY1 | NHEJ1 | NHLRC1 |
| NHP2 | NHS | NIDDM3 | NIDDM4 | NIN | NIPA1 | NIPAL4 | NIPBL |
| NKX2-1 | NKX2-5 | NKX2-6 | NKX3-2 | NKX6-2 | NLGN3 | NLGN4X | NLRC4 |
| NLRP1 | NLRP12 | NLRP3 | NLRP7 | NME1 | NME8 | NMNAT1 | NNMT |
| NNT | NOBOX | NOD2 | NODAL | NOG | NOL3 | NOLA2 | NOLA3 |
| NONO | NOP10 | NOP56 | NOS2 | NOS3 | NOTCH1 | NOTCH2 | NOTCH3 |
| NPC1 | NPC1L1 | NPC2 | NPHP1 | NPHP3 | NPHP4 | NPHS1 | NPHS2 |
| NPM1 | NPPA | NPR2 | NPRL2 | NPRL3 | NPS | NPSR1 | NQO1 |
| NQO2 | NR0B1 | NR0B2 | NR1H4 | NR2E3 | NR2F1 | NR2F2 | NR3C1 |
| NR3C2 | NR4A3 | NR5A1 | NRAS | NRG1 | NRL | NRXN1 | NSD1 |
| NSD3 | NSDHL | NSMCE2 | NSMCE3 | NSMF | NSUN2 | NT5C2 | NT5C3A |
| NT5E | NTF4 | NTHL1 | NTNG1 | NTRK1 | NTRK2 | NUBPL | NUDT15 |
| NUMA1 | NUP107 | NUP155 | NUP205 | NUP214 | NUP62 | NUP93 | NUS1 |
| NYX | OAT | OBSL1 | OCA2 | OCLN | OCRL | ODAPH | ODC1 |
| OFD1 | OGDH | OGG1 | OGT | OLR1 | OPA1 | OPA3 | OPCML |
| OPHN1 | OPLAH | OPN1LW | OPN1MW | OPN1SW | OPTN | ORAI1 | ORC1 |
| ORC4 | ORC6 | OSBPL2 | OSGEP | OSMR | OSTM1 | OTC | OTOA |
| OTOF | OTOG | OTOGL | OTUD6B | OTULIN | OTX2 | OVOL2 | OXCT1 |
| P2RX2 | P2RY12 | P3H1 | P3H2 | P4HA2 | P4HB | PABPN1 | PACS1 |
| PADI3 | PADI4 | PADI6 | PAFAH1B1 | PAH | PAK3 | PALB2 | PALLD |
| PAM16 | PANK2 | PAPSS2 | PARK7 | PARN | PATL2 | PAX1 | PAX2 |
| PAX3 | PAX4 | PAX5 | PAX6 | PAX7 | PAX8 | PAX9 | PBX1 |
| PC | PCAP | PCBD1 | PCCA | PCCB | PCDH15 | PCDH19 | PCK1 |
| PCK2 | PCLO | PCNA | PCNT | PCSK1 | PCSK9 | PCYT1A | PDCD1 |
| PDCD10 | PDE10A | PDE11A | PDE3A | PDE4D | PDE6A | PDE6B | PDE6C |
| PDE6D | PDE6G | PDE6H | PDE8B | PDGFB | PDGFRA | PDGFRB | PDGFRL |
| PDHA1 | PDHB | PDHX | PDIA3P1 | PDK3 | PDLIM4 | PDP1 | PDSS1 |
| PDSS2 | PDX1 | PDYN | PDZD7 | PDZK1P1 | PEPD | PER2 | PER3 |
| PET100 | PEX1 | PEX10 | PEX11B | PEX12 | PEX13 | PEX14 | PEX16 |
| PEX19 | PEX2 | PEX26 | PEX3 | PEX5 | PEX6 | PEX7 | PFKM |
| PFN1 | PGAM2 | PGAP1 | PGAP2 | PGAP3 | PGK1 | PGM1 | PGM3 |
| PGR | PHB | PHC1 | PHEX | PHF6 | PHF8 | PHGDH | PHIP |
| PHKA1 | PHKA2 | PHKB | PHKG2 | PHOX2A | PHOX2B | PHYH | PHYKPL |
| PI4KA | PIBF1 | PICALM | PIEZO1 | PIEZO2 | PIGA | PIGC | PIGG |
| PIGH | PIGL | PIGM | PIGN | PIGO | PIGP | PIGT | PIGV |
| PIGW | PIGY | PIH1D3 | PIK3CA | PIK3CD | PIK3R1 | PIK3R2 | PIK3R5 |
| PIKFYVE | PINK1 | PIP5K1C | PITPNM3 | PITX1 | PITX2 | PITX3 | PJVK |
| PKD1 | PKD1L1 | PKD2 | PKHD1 | PKLR | PKP1 | PKP2 | PLA2G2A |
| PLA2G5 | PLA2G6 | PLA2G7 | PLAA | PLAG1 | PLAGL1 | PLAT | PLAU |
| PLCB1 | PLCB4 | PLCD1 | PLCE1 | PLCG2 | PLCZ1 | PLD1 | PLD3 |
| PLEC | PLEKHG2 | PLEKHG5 | PLEKHM1 | PLG | PLIN1 | PLK4 | PLN |
| PLOD1 | PLOD2 | PLOD3 | PLP1 | PLPP6 | PLS3 | PMM2 | PMP22 |
| PMPCA | PMPCB | PMS2 | PMVK | PNKD | PNKP | PNLIP | PNP |
| PNPLA1 | PNPLA2 | PNPLA6 | PNPLA8 | PNPO | PNPT1 | POC1A | POC1B |
| POF1B | POFUT1 | POGLUT1 | POGZ | POLA1 | POLD1 | POLE | POLG |
| POLG2 | POLH | POLR1A | POLR1C | POLR1D | POLR3A | POLR3B | POMC |
| POMGNT1 | POMGNT2 | POMK | POMP | POMT1 | POMT2 | PON1 | POP1 |
| POR | PORCN | POT1 | POU1F1 | POU3F4 | POU4F3 | POU6F2 | PPA2 |
| PPARG | PPARGC1B | PPIB | PPM1D | PPM1K | PPOX | PPP1CB | PPP1R15B |
| PPP1R17 | PPP1R3A | PPP2R1A | PPP2R1B | PPP2R2B | PPP2R5D | PPP3CA | PPT1 |
| PQBP1 | PRCC | PRCD | PRDM12 | PRDM16 | PRDM5 | PRDM6 | PRDM8 |
| PRDX1 | PREPL | PRF1 | PRG4 | PRICKLE1 | PRIMPOL | PRKAB2 | PRKACA |
| PRKACG | PRKAG2 | PRKAR1A | PRKCD | PRKCG | PRKCH | PRKCSH | PRKD1 |
| PRKDC | PRKG1 | PRKN | PRKRA | PRLR | PRMT7 | PRNP | PROC |
| PRODH | PROK2 | PROKR2 | PROM1 | PROP1 | PROS1 | PROZ | PRPF3 |
| PRPF31 | PRPF4 | PRPF6 | PRPF8 | PRPH | PRPH2 | PRPS1 | PRRT2 |
| PRRX1 | PRSS1 | PRSS12 | PRSS56 | PRUNE1 | PRX | PSAP | PSAT1 |
| PSEN1 | PSEN2 | PSENEN | PSMA6 | PSMB4 | PSMB8 | PSMB9 | PSMC3IP |
| PSMD12 | PSPH | PSTPIP1 | PTCH1 | PTCH2 | PTCHD1 | PTDSS1 | PTEN |
| PTF1A | PTGDR | PTGER2 | PTGIS | PTH | PTH1R | PTHLH | PTPN1 |
| PTPN11 | PTPN12 | PTPN14 | PTPN22 | PTPRC | PTPRF | PTPRJ | PTPRO |
| PTPRQ | PTPRU | PTPRZ1 | PTRH2 | PTS | PUF60 | PUM1 | PURA |
| PUS1 | PUS3 | PXDN | PYCR1 | PYCR2 | PYGL | PYGM | PYROXD1 |
| QARS | QDPR | QRICH1 | RAB11B | RAB18 | RAB23 | RAB27A | RAB28 |
| RAB33B | RAB39B | RAB3GAP1 | RAB3GAP2 | RAB7A | RAC1 | RAC2 | RAD21 |
| RAD50 | RAD51 | RAD51C | RAD51D | RAD54B | RAD54L | RAF1 | RAG1 |
| RAG2 | RAI1 | RANBP2 | RAPSN | RARA | RARB | RARS | RARS2 |
| RASA1 | RASGRP2 | RASSF1 | RAX | RAX2 | RB1 | RB1CC1 | RBBP8 |
| RBCK1 | RBM10 | RBM12 | RBM15 | RBM20 | RBM28 | RBM8A | RBMX |
| RBP3 | RBP4 | RBPJ | RCBTB1 | RD3 | RDH11 | RDH12 | RDH5 |
| RDX | RECQL4 | REEP1 | REEP2 | RELB | RELN | REN | REPS1 |
| RERE | REST | RET | RETN | RETREG1 | RFT1 | RFWD3 | RFX5 |
| RFX6 | RFXANK | RFXAP | RGR | RGS5 | RGS9 | RGS9BP | RHAG |
| RHBDF2 | RHCE | RHD | RHO | RHOBTB2 | RIMS1 | RIN2 | RIPK4 |
| RIPOR2 | RIPPLY2 | RIT1 | RLBP1 | RLIM | RMND1 | RMRP | RNASEH1 |
| RNASEH2A | RNASEH2B | RNASEH2C | RNASEL | RNASET2 | RNF113A | RNF125 | RNF135 |
| RNF139 | RNF168 | RNF170 | RNF212 | RNF213 | RNF216 | RNF43 | RNF6 |
| RNU4ATAC | ROBO2 | ROBO3 | ROGDI | ROM1 | ROR1 | ROR2 | RORC |
| RP1 | RP1L1 | RP2 | RP9 | RPE65 | RPGR | RPGRIP1 | RPGRIP1L |
| RPIA | RPL10 | RPL11 | RPL15 | RPL21 | RPL26 | RPL27 | RPL35A |
| RPL5 | RPS10 | RPS14 | RPS17 | RPS19 | RPS23 | RPS24 | RPS26 |
| RPS27 | RPS28 | RPS29 | RPS6KA3 | RPS7 | RPSA | RRM2B | RS1 |
| RSPH1 | RSPH3 | RSPH4A | RSPH9 | RSPO1 | RSPO2 | RSPO4 | RSPRY1 |
| RTEL1 | RTN2 | RTN4IP1 | RTN4R | RTTN | RUBCN | RUNX1 | RUNX2 |
| RUSC2 | RXYLT1 | RYR1 | RYR2 | S1PR2 | SACS | SAG | SALL1 |
| SALL2 | SALL4 | SAMD9 | SAMD9L | SAMHD1 | SAR1B | SARDH | SARS |
| SARS2 | SASS6 | SATB2 | SBDS | SBF1 | SBF2 | SC5D | SCARB1 |
| SCARB2 | SCARF2 | SCGB3A2 | SCN10A | SCN11A | SCN1A | SCN1B | SCN2A |
| SCN2B | SCN3A | SCN3B | SCN4A | SCN4B | SCN5A | SCN8A | SCN9A |
| SCNN1A | SCNN1B | SCNN1G | SCO1 | SCO2 | SCP2 | SCYL1 | SCZD2 |
| SDC3 | SDCCAG8 | SDHA | SDHAF1 | SDHAF2 | SDHB | SDHC | SDHD |
| SDR9C7 | SEC23A | SEC23B | SEC24D | SEC61A1 | SEC63 | SECISBP2 | SELENON |
| SEMA3A | SEMA3E | SEMA4A | SEMA7A | SEPSECS | SEPT12 | SEPT9 | SERAC1 |
| SERPINA1 | SERPINA6 | SERPINA7 | SERPINB6 | SERPINB7 | SERPINB8 | SERPINC1 | SERPIND1 |
| SERPINE1 | SERPINF1 | SERPINF2 | SERPING1 | SERPINH1 | SERPINI1 | SET | SETBP1 |
| SETD2 | SETD5 | SETX | SF3B1 | SF3B4 | SFRP4 | SFTPA2 | SFTPB |
| SFTPC | SFXN4 | SGCA | SGCB | SGCD | SGCE | SGCG | SGOL1 |
| SGPL1 | SGSH | SH2B3 | SH2D1A | SH3BP2 | SH3GL1 | SH3PXD2B | SH3TC2 |
| SHANK2 | SHANK3 | SHH | SHOC2 | SHOX | SHPK | SHROOM4 | SI |
| SIAE | SIGMAR1 | SIK1 | SIL1 | SIM1 | SIN3A | SIPA1L3 | SIX1 |
| SIX3 | SIX5 | SIX6 | SKI | SKIV2L | SLC10A2 | SLC11A1 | SLC11A2 |
| SLC12A1 | SLC12A3 | SLC12A5 | SLC12A6 | SLC13A5 | SLC14A1 | SLC16A1 | SLC16A12 |
| SLC16A2 | SLC17A3 | SLC17A5 | SLC17A8 | SLC17A9 | SLC18A2 | SLC18A3 | SLC19A2 |
| SLC19A3 | SLC1A1 | SLC1A2 | SLC1A3 | SLC1A4 | SLC20A2 | SLC22A12 | SLC22A18 |
| SLC22A4 | SLC22A5 | SLC24A1 | SLC24A4 | SLC24A5 | SLC25A1 | SLC25A12 | SLC25A13 |
| SLC25A15 | SLC25A19 | SLC25A20 | SLC25A22 | SLC25A24 | SLC25A26 | SLC25A3 | SLC25A32 |
| SLC25A38 | SLC25A4 | SLC25A46 | SLC26A1 | SLC26A2 | SLC26A3 | SLC26A4 | SLC26A5 |
| SLC26A8 | SLC27A4 | SLC29A3 | SLC2A1 | SLC2A10 | SLC2A2 | SLC2A9 | SLC30A10 |
| SLC30A2 | SLC30A8 | SLC30A9 | SLC33A1 | SLC34A1 | SLC34A2 | SLC34A3 | SLC35A1 |
| SLC35A2 | SLC35A3 | SLC35C1 | SLC35D1 | SLC36A2 | SLC37A4 | SLC38A8 | SLC39A13 |
| SLC39A14 | SLC39A4 | SLC39A5 | SLC39A8 | SLC3A1 | SLC40A1 | SLC44A4 | SLC45A1 |
| SLC45A2 | SLC46A1 | SLC4A1 | SLC4A11 | SLC4A4 | SLC52A1 | SLC52A2 | SLC52A3 |
| SLC5A1 | SLC5A2 | SLC5A5 | SLC5A7 | SLC6A1 | SLC6A14 | SLC6A17 | SLC6A19 |
| SLC6A2 | SLC6A20 | SLC6A3 | SLC6A4 | SLC6A5 | SLC6A8 | SLC6A9 | SLC7A14 |
| SLC7A7 | SLC7A9 | SLC9A1 | SLC9A3 | SLC9A3R1 | SLC9A6 | SLC9A9 | SLCO1B1 |
| SLCO1B3 | SLCO2A1 | SLFN14 | SLITRK1 | SLITRK6 | SLURP1 | SLX4 | SMAD3 |
| SMAD4 | SMAD6 | SMAD7 | SMAD9 | SMARCA2 | SMARCA4 | SMARCAD1 | SMARCAL1 |
| SMARCB1 | SMARCD2 | SMARCE1 | SMC1A | SMC3 | SMCHD1 | SMCP | SMG9 |
| SMIM1 | SMN1 | SMN2 | SMOC1 | SMOC2 | SMPD1 | SMPX | SMS |
| SNAI2 | SNAP25 | SNAP29 | SNCA | SNCB | SNIP1 | SNRNP200 | SNRPB |
| SNRPE | SNRPN | SNTA1 | SNX10 | SNX14 | SOBP | SOD1 | SOD2 |
| SOHLH1 | SON | SORD | SORT1 | SOS1 | SOS2 | SOST | SOX10 |
| SOX11 | SOX17 | SOX18 | SOX2 | SOX3 | SOX5 | SOX9 | SP110 |
| SP7 | SPAG1 | SPARC | SPAST | SPATA16 | SPATA5 | SPATA7 | SPECC1L |
| SPEG | SPG11 | SPG20 | SPG21 | SPG7 | SPINK1 | SPINK5 | SPINT2 |
| SPR | SPRED1 | SPRTN | SPRY2 | SPRY4 | SPTA1 | SPTAN1 | SPTB |
| SPTBN2 | SPTBN4 | SPTLC1 | SPTLC2 | SQSTM1 | SRC | SRCAP | SRD5A2 |
| SRD5A3 | SRGAP1 | SRP72 | SRPX2 | SRY | SSR4 | SSX1 | SSX2 |
| ST14 | ST3GAL3 | ST3GAL5 | STAC3 | STAG1 | STAG3 | STAMBP | STAR |
| STAT1 | STAT2 | STAT3 | STAT4 | STAT5B | STEAP3 | STIL | STIM1 |
| STK11 | STK4 | STN1 | STOX1 | STRA6 | STRADA | STRC | STS |
| STT3A | STT3B | STUB1 | STX11 | STX16 | STX1B | STXBP1 | STXBP2 |
| SUCLA2 | SUCLG1 | SUFU | SUGCT | SULT2B1 | SUMF1 | SUMO1 | SUMO4 |
| SUN5 | SUOX | SURF1 | SYCE1 | SYCP3 | SYN1 | SYN2 | SYNE1 |
| SYNE2 | SYNE4 | SYNGAP1 | SYNJ1 | SYP | SYT14 | SYT2 | SZT2 |
| TAB2 | TAC3 | TACO1 | TACR3 | TACSTD2 | TAF1 | TAF12 | TAF13 |
| TAF15 | TAF2 | TAF4B | TAF6 | TAL1 | TAL2 | TALDO1 | TANGO2 |
| TAP1 | TAP2 | TAPBP | TAPT1 | TARDBP | TARS2 | TAS2R16 | TAS2R38 |
| TAT | TAZ | TBC1D20 | TBC1D23 | TBC1D24 | TBC1D4 | TBC1D7 | TBCD |
| TBCE | TBCK | TBK1 | TBL1XR1 | TBP | TBX1 | TBX15 | TBX18 |
| TBX19 | TBX20 | TBX21 | TBX22 | TBX3 | TBX4 | TBX5 | TBX6 |
| TBXA2R | TBXAS1 | TCAP | TCF12 | TCF3 | TCF4 | TCF7L2 | TCHH |
| TCIRG1 | TCL1A | TCL1B | TCN2 | TCOF1 | TCTEX1D2 | TCTN1 | TCTN2 |
| TCTN3 | TDGF1 | TDO2 | TDP1 | TDP2 | TDRD7 | TEAD1 | TECPR2 |
| TECR | TECRL | TECTA | TEK | TELO2 | TENM3 | TENM4 | TERC |
| TERT | TET2 | TEX11 | TEX14 | TEX15 | TF | TFAM | TFAP2A |
| TFAP2B | TFE3 | TFG | TFR2 | TFRC | TG | TGDS | TGFB1 |
| TGFB2 | TGFB3 | TGFBI | TGFBR1 | TGFBR2 | TGIF1 | TGM1 | TGM3 |
| TGM5 | TGM6 | TH | THAP1 | THBD | THBS2 | THOC2 | THOC6 |
| THPO | THRA | THRB | TIA1 | TICAM1 | TIMM50 | TIMM8A | TIMMDC1 |
| TIMP3 | TINF2 | TIRAP | TJP2 | TK2 | TKT | TLE6 | TLL1 |
| TLR1 | TLR2 | TLR3 | TLR5 | TM4SF20 | TMC1 | TMC6 | TMC8 |
| TMCO1 | TMEM106B | TMEM107 | TMEM126A | TMEM126B | TMEM127 | TMEM138 | TMEM165 |
| TMEM173 | TMEM199 | TMEM216 | TMEM231 | TMEM237 | TMEM240 | TMEM260 | TMEM38B |
| TMEM43 | TMEM67 | TMEM70 | TMEM98 | TMIE | TMLHE | TMPRSS15 | TMPRSS3 |
| TMPRSS6 | TMTC3 | TNC | TNF | TNFAIP3 | TNFRSF10B | TNFRSF11A | TNFRSF11B |
| TNFRSF13B | TNFRSF13C | TNFRSF1A | TNFRSF4 | TNFSF11 | TNFSF4 | TNIK | TNNC1 |
| TNNI2 | TNNI3 | TNNI3K | TNNT1 | TNNT2 | TNNT3 | TNPO3 | TNXB |
| TOE1 | TOPORS | TOR1A | TOR1AIP1 | TP53 | TP53RK | TP63 | TP73 |
| TPCN2 | TPH2 | TPI1 | TPK1 | TPM1 | TPM2 | TPM3 | TPMT |
| TPO | TPP1 | TPRKB | TPRN | TRAC | TRAF3 | TRAF3IP1 | TRAF3IP2 |
| TRAIP | TRAPPC11 | TRAPPC12 | TRAPPC2 | TRAPPC6B | TRAPPC9 | TRDN | TREH |
| TREM2 | TREX1 | TRH | TRIM2 | TRIM32 | TRIM33 | TRIM36 | TRIM37 |
| TRIM44 | TRIO | TRIOBP | TRIP11 | TRIP12 | TRIP13 | TRIP4 | TRIT1 |
| TRMT10A | TRMT10C | TRMT5 | TRMU | TRNT1 | TRPA1 | TRPC3 | TRPC6 |
| TRPM1 | TRPM4 | TRPM6 | TRPM7 | TRPS1 | TRPV3 | TRPV4 | TSC1 |
| TSC2 | TSEN15 | TSEN2 | TSEN34 | TSEN54 | TSFM | TSGA10 | TSHB |
| TSHR | TSHZ1 | TSPAN12 | TSPAN7 | TSPEAR | TSPYL1 | TSR2 | TTBK2 |
| TTC19 | TTC21B | TTC25 | TTC37 | TTC7A | TTC8 | TTI2 | TTLL5 |
| TTN | TTPA | TTR | TUB | TUBA1A | TUBA3D | TUBA4A | TUBA8 |
| TUBB | TUBB1 | TUBB2A | TUBB2B | TUBB3 | TUBB4A | TUBB4B | TUBB6 |
| TUBB8 | TUBG1 | TUBGCP4 | TUBGCP6 | TUFM | TULP1 | TUSC3 | TWIST1 |
| TWIST2 | TWNK | TXN2 | TXNL4A | TXNRD2 | TYK2 | TYMP | TYR |
| TYROBP | TYRP1 | UBA1 | UBA5 | UBB | UBE2A | UBE2T | UBE3A |
| UBE3B | UBIAD1 | UBQLN2 | UBR1 | UBTF | UCHL1 | UCP1 | UCP2 |
| UCP3 | UFM1 | UFSP2 | UGT1A1 | UGT2B17 | UMOD | UMPS | UNC119 |
| UNC13D | UNC45B | UNC80 | UNC93B1 | UNG | UPB1 | UPF3B | UQCC2 |
| UQCC3 | UQCRB | UQCRC2 | UQCRQ | UROC1 | UROD | UROS | USB1 |
| USF1 | USH1C | USH1G | USH1H | USH2A | USP18 | USP27X | USP48 |
| USP8 | USP9X | USP9Y | UVSSA | VAC14 | VAMP1 | VANGL1 | VANGL2 |
| VAPB | VARS | VARS2 | VAX1 | VCAM1 | VCAN | VCL | VCP |
| VDR | VEGFA | VEGFC | VHL | VIM | VIPAS39 | VKORC1 | VLDLR |
| VMA21 | VPS11 | VPS13A | VPS13B | VPS13C | VPS33A | VPS33B | VPS35 |
| VPS37A | VPS45 | VPS53 | VRK1 | VSX1 | VSX2 | VWA3B | VWF |
| WAC | WARS | WARS2 | WAS | WASHC4 | WASHC5 | WBP2 | WDFY3 |
| WDPCP | WDR11 | WDR19 | WDR26 | WDR34 | WDR35 | WDR36 | WDR45 |
| WDR45B | WDR60 | WDR62 | WDR72 | WDR73 | WDR81 | WEE2 | WFS1 |
| WHRN | WIPF1 | WISP3 | WNK1 | WNK4 | WNT1 | WNT10A | WNT10B |
| WNT3 | WNT4 | WNT5A | WNT7A | WRAP53 | WRN | WT1 | WWC1 |
| WWOX | XBP1 | XDH | XIAP | XIST | XK | XPA | XPC |
| XPNPEP2 | XPNPEP3 | XPR1 | XRCC1 | XRCC2 | XRCC3 | XRCC4 | XYLT1 |
| XYLT2 | YAP1 | YARS | YARS2 | YME1L1 | YWHAG | YY1 | YY1AP1 |
| ZAP70 | ZBTB16 | ZBTB18 | ZBTB20 | ZBTB24 | ZBTB42 | ZC3H14 | ZC4H2 |
| ZDHHC15 | ZDHHC9 | ZEB1 | ZEB2 | ZFHX2 | ZFHX3 | ZFHX4 | ZFP57 |
| ZFPM2 | ZFYVE26 | ZFYVE27 | ZIC1 | ZIC2 | ZIC3 | ZMPSTE24 | ZMYND10 |
| ZMYND11 | ZMYND15 | ZNF141 | ZNF148 | ZNF335 | ZNF365 | ZNF408 | ZNF423 |
| ZNF469 | ZNF513 | ZNF644 | ZNF687 | ZNF711 | ZNF750 | ZNHIT3 | ZP1 |
| ZP3 | ZSWIM6 |  |  |  |  |  |  |
